# Lamina‐specific properties of spinal astrocytes

**DOI:** 10.1002/glia.23990

**Published:** 2021-03-10

**Authors:** Mira T. Kronschläger, Anna S. M. Siegert, Felix J. Resch, Pradeep S. Rajendran, Baljit S. Khakh, Jürgen Sandkühler

**Affiliations:** ^1^ Department of Neurophysiology, Center for Brain Research Medical University of Vienna Vienna Austria; ^2^ Department of Physiology, David Geffen Schoof of Medicine University of California Los Angeles Los Angeles California USA; ^3^ UCLA Cardiac Arrhythmia Center, Neurocardiology Research Program of Excellence, David Geffen School of Medicine University of California Los Angeles Los Angeles California USA

**Keywords:** astrocytes, diversity, functional adaptation, nociception, spinal cord, upper dorsal horn

## Abstract

Astrocytes are indispensable for proper neuronal functioning. Given the diverse needs of neuronal circuits and the variety of tasks astrocytes perform, the perceived homogeneous nature of astrocytes has been questioned. In the spinal dorsal horn, complex neuronal circuitries regulate the integration of sensory information of different modalities. The dorsal horn is organized in a distinct laminar manner based on termination patterns of high‐ and low‐threshold afferent fibers and neuronal properties. Neurons in laminae I (L1) and II (L2) integrate potentially painful, nociceptive information, whereas neurons in lamina III (L3) and deeper laminae integrate innocuous, tactile information from the periphery. Sensory information is also integrated by an uncharacterized network of astrocytes. How these lamina‐specific characteristics of neuronal circuits of the dorsal horn are of functional importance for properties of astrocytes is currently unknown. We addressed if astrocytes in L1, L2, and L3 of the upper dorsal horn of mice are differentially equipped for the needs of neuronal circuits that process sensory information of different modalities. We found that astrocytes in L1 and L2 were characterized by a higher density, higher expression of GFAP, Cx43, and GLAST and a faster coupling speed than astrocytes located in L3. L1 astrocytes were more responsive to Kir4.1 blockade and had higher levels of AQP4 compared to L3 astrocytes. In contrast, basic membrane properties, network formation, and somatic intracellular calcium signaling were similar in L1–L3 astrocytes. Our data indicate that the properties of spinal astrocytes are fine‐tuned for the integration of nociceptive versus tactile information.

## Main Points

- In‐depth characterization of spinal astrocytes in the upper dorsal horn of mice.
- Distinct lamina‐ and circuit‐specific properties of spinal astrocytes.
- Fine‐tuning of astrocytic properties for the integration of nociceptive versus tactile information.

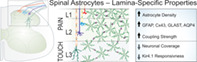

## 1 INTRODUCTION

Tasked with performing multitude physiological responses, astrocytes are indispensable for proper neuronal functioning (for review see Sofroniew & Vinters, 2010; Ransom & Ransom, 2012; Nedergaard, Ransom, & Goldman, 2003; Verkhratsky & Nedergaard, 2014; Verkhratsky, Parpura, & Kettenmann, 2014). Astrocytes tile the entire central nervous system (CNS), providing essential support to neurons: a single astrocyte thereby ensheaths ~100,000 synapses (Bushong, Martone, Jones, & Ellisman, 2002; Chai et al., 2017). At the synaptic level, astrocytes maintain homeostasis by buffering excess ions and neurotransmitters after neuronal activity while also regulating overall osmotic pressure by guiding water‐ and glymphatic clearance (for review see Mahmoud, Gharagozloo, Simard, & Gris, 2019; Jessen, Munk, Lundgaard, & Nedergaard, 2015). Astrocytes provide metabolic and energetic support to neurons (for review see Allaman, Bélanger, & Magistretti, 2011) and modulate neuronal plasticity (Kronschläger et al., 2016; Henneberger, Papouin, Oliet, & Rusakov, 2010, for review see Xanthos & Sandkühler, 2014). Further, being considered as immune competent cells, astrocytes participate in orchestrating responses to elevated neuronal activity, pathogens or injury (for review see Colombo & Farina, 2016; Dong & Benveniste, 2001).

Despite this diversity in tasks and the diverse needs of neuronal circuits, astrocytes were long considered as homogenous—until the concept of interregional heterogeneity of astrocytes was shown in supraspinal regions of the CNS and paved the way for an appreciation of the complexity of this cell population (Chai et al., 2017; John Lin et al., 2017; Lozzi, Huang, Sardar, Huang, & Deneen, 2020), for review see (Khakh & Deneen, 2019).

The spinal cord too is a CNS structure with highly diverse regional properties and functions, possibly demanding distinct types of astrocytes. An in‐depth characterization of astrocytic features and properties, however, does not exist. To date, the investigation of spinal astrocytes has focused on a developmental (Tsai et al., 2012) or broad regional diversity between dorsal/ventral laminae (Molofsky et al., 2014) and little is known about detailed phenotypic characteristics of spinal astrocytes (but see Kohro et al., 2020). Spinal neurons coordinate the transfer of information from and to the CNS and thereby represent an important level for processing information about the environment and the interaction with it. Whereas the ventral horn of the spinal cord carries efferent motor information to the periphery, the dorsal horn integrates all somatosensory information from the periphery involving a complex neuronal circuitry as well as a yet uncharacterized network of astrocytes.

At the spinal dorsal horn, sensory information of different modalities is integrated in a concise laminar manner. This laminar organization is stipulated by the termination pattern of primary afferent fibers for different sensory modalities and postsynaptic properties of spinal neurons (Molander, Xu, & Grant, 1984; Rexed, 1952). The superficial laminae L1 and L2 are primarily targeted by high‐threshold sensory fibers relaying information about noxious, potentially painful or damaging stimuli to second‐order neurons—forming the nociceptive circuits of the dorsal horn. L1 therein delineates the border to the white matter and holds the majority of projection neurons with trajectories to supraspinal regions. L1 is mostly targeted by unmyelinated, peptidergic C‐fibers and lightly myelinated Aδ‐fibers. L2 is fostered by interneurons only and represents the major termination zone for nonpeptidergic C‐fibers. L3 and deeper laminae, on the other hand, are targeted by low‐threshold afferents relaying information about nonnoxious sensory information such as light touch, pressure and proprioception—the myelinated Aβ‐fibers. Along with deeper laminae, neurons in L3 form the tactile circuits of the dorsal horn with their projection neurons residing at the border to L4 or deeper laminae.

Whether excited by high‐ or low‐threshold stimuli, all primary afferent sensory neurons are glutamatergic in nature. Increasing stimuli intensities are thereby associated with higher levels of extracellular glutamate and K^+^ ions in the dorsal horn (Al‐Ghoul, Li, Weinberg, & Rustioni, 1993; Heinemann, Schaible, & Schmidt, 1990; Kangrga & Randic, 1991). In the upper dorsal horn, incoming noxious and innocuous signals are integrated by excitatory and inhibitory interneurons that—under control of descending modulation—regulate the output of projection neurons for supraspinal processing (for review see Todd, 2010). In the superficial laminae L1 and L2, 70% of the second‐order neurons are excitatory interneurons, whereas L3 is distinguished by holding the majority of inhibitory interneurons. While GABAergic interneurons are more evenly distributed across the upper dorsal horn, the proportion of glycinergic neurons is markedly higher in L3 compared to L1 and L2 (Polgár et al., 2003; Polgár, Durrieux, Hughes, & Todd, 2013; Todd & Sullivan, 1990). Further, the overall neuronal density is said to be higher in L1 and L2 compared to L3 (for review see Peirs, Dallel, & Todd, 2020).

Thus, the upper dorsal horn L1–L3 constitutes a highly diverse neuronal environment with different requirements for homeostatic regulations such as neurotransmitter metabolism, ion homeostasis and overall support. In close spatial proximity, astrocytes in L1–L3 have to serve the diverse claims of their respective neuronal environment. The modality‐specific wiring of neuronal circuits in the upper dorsal horn likely demand region‐specific properties of spinal astrocytes. This hypothesis has, however, not yet been tested. Notably, the distinct topographical organization of the upper dorsal horn allowed us to address a circuit‐ and lamina‐specific heterogeneity of spinal astrocytes within a narrow spatial allocation, which, as far as we know, has not been achieved for other areas of the nervous system.

In this study, we addressed if and how spinal astrocytes are differentially equipped for the processing of sensory information of different modalities. In an effort to fill substantial gaps in the understanding of the overall capacity of astrocytes, we aimed to identify their diverse nature and the potential adaptations to neuronal circuitries at the spinal level.

## 2 METHODS

### 2.1 Animals

Experiments were carried out at the University of California, Los Angeles (UCLA) and the Medical University of Vienna, Austria (MUW). All animal experiments were conducted in accordance with the National Institute of Health Guide for the Care and Use of Laboratory Animals and the European Community's Council directives 2010/63/EU. All animals and experiments were approved by the Chancellor's Animal Research Committee at the University of California, Los Angeles and by the Austrian Federal Ministry for Education, Science and Culture (BMBWF‐66.009/0172‐V/3b/2018). All experiments were performed according to the rules of the Good Scientific Practice Guide of the MUW. All mice were housed with food and water available ad libitum in a 12 hr light/dark environment. Humidity (~60%) and temperature (~21°C) were monitored and controlled. Housing enrichment was provided at all times. Data for experiments were collected from male and female adult mice (7–14 weeks).

### 2.2 Mouse lines

Aldh1l1‐eGFP mice (MMRRC #3843271; 10‐formyltetrahydrofolate dehydrogenase‐enhanced green fluorescent protein) on a Swiss‐Webster background were acquired from MMRRC and maintained by breeding with Swiss‐Webster mice (from Taconic Biosciences). Breeding and processing were performed at UCLA. Aldh1l1‐CreER^T2^ (JAX 029655; cre recombinase mutant human estrogen receptor T2) and Ai95 mice (JAX 028865) were purchased from Jackson Laboratory via Charles River and maintained by breeding with wild‐type C57Bl/6N or C57Bl/6J mice, respectively. Breeding and processing was performed at the MUW. Heterozygous double transgenic mice and wild‐type littermates were used for experiments. Wild‐type C57Bl/6 were additionally purchased from the Department of Laboratory Animal Science and Genetics of the Medical University of Vienna (Himberg, Austria). To induce full expression of the calcium indicator GCaMP6f, Aldh1l1‐CreER^T2^ × Ai95 mice were injected intraperitoneally (i.p.) with daily 75 mg/kg Tamoxifen per mouse (100 μl from a 20 mg/ml stock; Sigma T5648; dissolved in corn oil, Sigma C8267) for 5 consecutive days. For sparse expression of the GCaMP6f, a single i.p. injection of 75 mg/kg Tamoxifen per mouse was used. All animals were closely monitored during and for 5 days after injections. Experiments were performed 14 days after the last injection.

### 2.3 Sca*l*eS—Optical clearing of spinal cord sections

Tissue clearing using the Sca*l*eS method was performed as described previously (Hama et al., 2015). For transcardial perfusion, Aldh1l1‐eGFP mice were injected with heparin to prevent blood clotting (100 units, i.p.) and then euthanized with pentobarbital overdose. After loss of all reflexes, a thoracotomy was performed and mice were perfused with 50 ml of ice‐cold 0.01 M phosphate‐buffered saline (1× PBS), followed by 50 ml of ice‐cold 4% paraformaldehyde (PFA; Electron Microscopy Sciences). The spinal cord was carefully removed from the vertebral column and postfixed overnight at +4°C. One millimeter‐thick transversal sections of the lumbar spinal cord were cut manually using sharp razor blades. Spinal cord slices were then cleared using the Sca*l*eS protocol to allow for deeper imaging of endogenous fluorescence while preserving the three‐dimensional architecture. In short, sections were incubated in Sca*l*eSQ(5) (22.5% D‐(‐)‐sorbitol [w/v], 9.1 M urea and 5% Triton X‐100 [w/v] in distilled water; pH 8.2) for 2 hr at 37°C under gentle agitation. Samples were then mounted overnight in Sca*l*eS4(0) (40% D‐(‐)‐sorbitol [w/v], 10% glycerol [w/v], 4 M urea and 15% dimethylsulfoxide [v/v] in distilled water; pH 8.1; refractive index 1.437). Cleared slices were imaged using a Zeiss LSM 780 confocal microscope, and semi‐automated cell counting of astrocytes was performed using Imaris (Bitplane V9.6.0).

### 2.4 Immunohistochemical staining

For all Immunohistochemical (IHC) staining, mice were euthanized by pentobarbital overdose (200 mg/kg; Richter Pharma AG). After loss of all reflexes, mice were perfused transcardially with ice cold 0.9% NaCl containing 0.1% heparin (Braun, 2047217), followed by 4% PFA (pH 7.4). The whole lumbar segment of the spinal cord was removed and postfixed in 4% PFA overnight at +4°C. For cryoprotection, the spinal cords were placed in 0.1 M phosphate buffer solution with 20% sucrose for 24 hr followed by 30% sucrose 0.1 M phosphate buffer solution for another 24 hr at +4°C. Afterwards, the spinal cord samples were embedded in optimal cutting temperature compound (OCT; Sakura Finetek) and stored at −80°C until further use. PFA, 1× PBS and 0.1 M phosphate buffer were always freshly prepared.

The spinal cords were then sliced using a cryotome (Leica, CM3050 S) collecting all lumbar sections (L1–L5). The slices were stored in 1× PBS + NaN_3_ (0.05%; Sigma S‐2002) at +4°C until further use. The thickness of the slices was dependent on the experimental set‐up. For analysis of expression pattern, 40 μm thick slices were used. For coverage analysis, 150 μm thick slices were produced to cover the whole surrounding of single astrocytes.

Free‐floating indirect IHC stainings were performed. Tissue slices were washed in 1× PBS (×3 10 min) and then incubated for 40 min in blocking solution comprising 1× PBS, 0.5% Triton X‐100 (Merck, 9036‐19‐5) and 10% normal goat serum (NGS; Thermo Fisher Scientific 10000C) on a shaker at room temperature. Afterwards, primary antibodies were added in a 1× PBS solution containing 0.05% Triton X‐100 and 1% NGS on a shaker for 60 min, and then incubated overnight at +4°C. On the next day, the slices were washed with 1× PBS (×3 10 min) before the secondary antibodies were added in a 1× PBS solution containing 0.05% Triton X‐100 and 1% NGS. The slices were incubated for 2 hr on a shaker at room temperature protected from light by foil. Slices were mounted on glass slides (Superfrost plus, Thermo Fisher Scientific J1800AMNT) using Fluoromount‐G mounting medium with 4′,6‐diamidino‐2‐phenylindole (DAPI; Thermo Fisher Scientific 00‐4959‐52) to stain cell nuclei. Glass slides were kept at +4°C at all times.

For the coverage analysis, a prolonged staining protocol was applied to guarantee antibody penetration. Solutions and washing times were identical. The blocking solution was applied for 60 min. The primary antibodies were allowed to incubate for 4 hr on a shaker at room temperature and then incubated for >50 hr at +4°C. The secondary antibodies were allowed to incubate for 4 hr on a shaker at room temperature and then incubated for >24 hr at +4°C always covered with foil. Afterwards, slices were incubated in a DAPI‐staining solution (20 μM; Abcam ab228549) for 2 hr on a shaker. Slices were washed ×3 in 1× PBS for 15 min before mounting on glass sides using Fluoromount‐G mounting medium (Thermo Fisher Scientific 00‐4958‐02). Glass slides were kept at 4°C at all times.

The following primary antibodies were used: isolectin B4 (IB4; 1:500; biotin‐conjugated, Vector B1205), anti‐protein kinase Cγ (PKCγ; 1:200; guinea pig, Frontier Institute PKCg‐GP‐Af350), anti‐calcitonin‐gene‐related‐peptide (CGRP; 1:1000; rabbit, Peninsula Labs T‐4032), anti‐GFP (1:1000; chicken, Thermo Fisher Scientific A10262), anti‐neuronal nuclei (NeuN; 1:1000; rabbit, Cell signaling 12943), anti‐glial fibrillary acidic protein (GFAP; 1:500; mouse, Merck Millipore MAB360/rabbit, Cell signaling 12389S), anti‐S100 calcium‐binding protein B (S100B; 1:2000; rabbit, Abcam ab52642), anti‐glutamine synthetase (GS; 1:2000; mouse, Merck Millipore MAB302), anti‐N‐myc downregulated gene 2 (NDRG2; 1:450; rabbit, Atlas Antibodies HPA002896), anti‐connexin 30 (Cx30; 1:500; rabbit, Abcam ab200866), anti‐Cx43 (1:1500; Abcam ab11370), anti‐excitatory amino acid transporter 1 (EAAT1; 1:100; rabbit, Abcam ab416; glutamate aspartate transporter‐1; GLAST), anit‐EAAT2 (1:3000; rabbit, Abcam ab41621; glutamate transporter‐1, GLT1), anti‐Kir4.1 (1:500; rabbit, Alomone labs APC‐035), and anti‐aquaporin 4 (AQP4; 1:100; rabbit, Sigma Aldrich A5971).

The following secondary antibodies were used: streptavidin‐conjugated Alexa Fluor 555 (1:1000; Thermo Fisher Scientific S32355), streptavidin‐conjugated Alexa Fluor 647 (1:2000; Thermo Fisher Scientific S21374), goat anti‐guinea pig Alexa Fluor 647 (1:250; Thermo Fisher Scientific A21450), goat anti‐mouse Alexa Fluor 488 (1:1500; Thermo Fisher Scientific A10680), goat anti‐mouse Alexa Fluor 546 (1:1500; Thermo Fisher A11003), and goat anti‐rabbit Alexa Fluor 488 (1:1000; Thermo Fisher Scientific A11008).

Fluorescent images were captured using an inverted confocal microscope (Leica TCS SP5 Confocal Laser Scanning Microscope, HCX PL APO CS ×20/0.70 NA glycerol immersion objective). 15–20 μm z‐stacks at 1 μm step size were acquired from one dorsal horn per slice. 4–6 slices were captured per mouse and the mean intensity per region of interest (ROI; i.e., sum of grey values of all pixels divided by the number of pixels) was calculated per mouse. Magnifications were acquired using a ×3 digital zoom. The excitation laser lines used were 405, 488, 561, and 633 nm, the pinhole was set at airy disc (60.67 μm). For fluorescence detection, photo multiplier tubes (PMT) and hybrid detectors (HyD) were used. Laser and detection settings were kept identical within each experiment (i.e., per marker for all slices and mice used). Images were analyzed using Fiji ImageJ (V1.53c).

For all IHC staining, PKCγ (marker for L2 inner) and IB4 (marker for L2 outer) were used to identify L2. Combining the DAPI stain and landmarks, L1 and L3 were defined. In addition, CGRP labelling was used for exemplary illustrations of L1. The lamina‐specific ROIs were drawn for each slice and only afterwards projected on the staining for the protein of interest to avoid any pattern‐based bias. Mean intensity unit (au; arbitrary unit) was measured for each ROI. All analyses were performed on raw images without any form of editing. Additionally, the area of each ROI was measured to ensure uniformity across multiple samples (data not shown). Images were only post hoc processed for exemplary purposes.

For imaging of single astrocytes, fluorescent images were captured using a HCX PL APO ×40/1.25 NA oil immersion objective with a ×5 digital zoom. Only single astrocytes with no overlapping regions with neighboring astrocytes and >10 μm distance in all xyz‐directions were used for analysis. z‐stacks at 0.5 μm step size were acquired. The astrocyte surface area was measured from a low‐intensity threshold reconstruction (surface smoothing 0.75 μm) encompassing the cell volume and the space between its processes. NeuN^+^ cells and DAPI^+^ cells with their somatic center within a 10 μm radius from the astrocytic surface area were semi‐automatically counted. Analysis was performed using Imaris (Bitplane; V9.6.0.).

### 2.5 Acute spinal cord slice preparation

For preparation of acute transversal spinal cord slices, mice were sacrificed by decapitation under deep isoflurane anesthesia once all reflexes subsided. The spinal cord was exposed by laminectomy and transferred into ice‐cold pre‐oxygenated incubation solution of following composition (in mM): 194 sucrose, 30 NaCl, 4.5 KCl, 1 MgCl_2_, 26 NaHCO_3_, 1.2 NaH_2_PO_4_, and 10 D‐glucose and was oxygenated with a 95% O_2_ and 5% CO_2_ mixture; pH 7.4, measured osmolarity 310–320 mosmol·l^−1^. The dura mater, ventral and dorsal roots were removed. The spinal cord was ventrally glued to an upright agar block and placed in a slicing chamber with ice‐cold pre‐oxygenated incubation solution. 300–400 μm thick transversal spinal cord slices (lumbar segments L1–L5) were cut on a microslicer (DTK‐1000, Dosaka, Kyoto, Japan). The slices were incubated for 30 min at 32°C and then stored at room temperature (~21°C) in oxygenated recording solution (standard artificial cerebrospinal fluid; aCSF). The recording solution consisted of (in mM): 124 NaCl, 4.5 KCl, 2 CaCl_2_, 1 MgCl_2_, 26 NaHCO_3_, 1.2 NaH_2_PO_4_, and 10 D‐glucose and was oxygenated with a 95% O_2_ and 5% CO_2_ mixture; pH 7.4, measured osmolarity 310–320 mosmol·l^−1^. Acute transversal spinal cord slices were used for all electrophysiological and imaging experiments.

### 2.6 Electrophysiology

A single slice was transferred to the recording chamber, fixed with a grid and allowed to acclimate for >5 min before any intervention. Continuous superfusion of oxygenated recording solution was maintained at a rate of 3.5 ml/min in an open system at all times. All recordings were performed at room temperature. Astrocytes of the dorsal horn were visualized with Dodt infrared optics (Dodt, Eder, Frick, & Zieglgänsberger, 1999) using a HCX APO L ×20/1.0 NA objective on a Leica DM6000CFS microscope. The bright luminescent band of the substantia gelatinosa was identified. L1 was defined as the area located within a distance of <30 μm to the white matter and the area dorsal to the substantia gelatinosa. L2 was defined as the region within the substantia gelatinosa. L3 was defined as the region located ventrally to the substania gelatinosa and with the use of landmarks. Astrocytes were recorded in whole‐cell patch‐clamp configuration with glass pipettes (4–6 MΩ) filled with intracellular solution consisting of (in mM): 135 K‐gluconate, 3 KCl, 10 HEPES, 1 EGTA, 0.3 Na_2_‐ATP, 4 Mg‐ATP, 0.1 CaCl_2_, 8 Na_2_‐phosphocreatine, pH 7.28 adjusted with KOH, measured osmolarity 295–300 mosmol·l^−1^. The intracellular solution was always filtered before use. Patch pipettes were pulled on a horizontal micropipette puller (P‐1000, Sutter Instruments, Novato, California) from borosilicate glass (Hilgenberg GmbH, Malsfeld, Germany). The resting membrane potential (RMP) was measured immediately after establishing whole‐cell configuration. Voltage‐clamp recordings were made at a holding potential of −70 mV using an Axopatch 200B patch‐clamp amplifier and the pCLAMP 10 acquisition software package (both Molecular Devices, Union City, California). Signals were sampled at 10 kHz. Data was analyzed offline using pCLAMP 10. Liquid junction potential of −15.6 mV was calculated and corrected. Series resistance (*R*_*s*_), total resistance (*R*_total_) and membrane resistance (*R*_*m*_ = *R*_total_ − *R*_*s*_) were calculated from the averaged reaction to 20 consecutive hyperpolarizing voltage steps from −70 to −80 mV for 100 ms. Current–voltage relationship (IV‐curves) were analyzed from 23 voltage steps of 10 mV step size each, starting from −160 to +60 mV. A depolarizing pre‐pulse to 0 mV was applied to monitor *R*_*s*_. To quantify the linearity, the rectification index (RI) was calculated as the ratio of the conductance 90 mV positive/negative to the reversal potential (RI = [−*y*1]/*y*2). The slope conductance was calculated in Excel. All passive membrane properties were recorded directly after establishing whole‐cell configuration and again 5 min after bath application of BaCl_2_ (BA; 100 μM; Merck 1.01717) or normal aCSF. An open superfusion system was maintained at all times. All electrophysiologcal data obtained from astrocytes have to be interpreted with due diligence and caution. The inherent low *R*_*m*_ of astrocytes creates a natural barrier to voltage‐clamp the membrane potential with high fidelity (Ma, Xu, Wang, Enyeart, & Zhou, 2014). Since the mean *R*_*s*_ was well below the recommended maximum *R*_*s*_ of 10 MΩ within and for all groups during all time points (total mean of 7.7 ± 0.2 MΩ) and all recordings were performed with identical intra‐ and extracellular solutions at matched time points, we report the comparisons bearing these limitations in mind. These empirical data are useful for comparative purposes between groups reported herein under identical conditions, but should be cautiously interpreted with regards to other studies that may have been performed under different conditions.

### 2.7 Network extension over time and real‐time gap junction coupling

A single slice was transferred to the recording chamber, fixed with a grid and allowed to acclimate for >5 min before any intervention. Continuous superfusion with oxygenated recording solution was maintained at a rate of 3.5 ml/min in an open system at all times. All recordings were performed at room temperature. To investigate network extension over time and gap junction coupling in real‐time, 0.1 mg/ml Sulforhodamine B (SRB; Sigma 341738) was added to the intracellular solution. Fluorescent images were acquired via multiphoton imaging on a Leica DM6000CFS microscope equipped with a ×20 objective (Leica HCX APO, NA 1.0) and a Chameleon‐XR Ti‐sapphire laser (Coherent, Inc.). SRB was excited at 820 nm, and fluorescence emission was collected with nondescanned detectors (NDDs) at 565–605 nm. Astrocytes were held in whole‐cell configuration and identified based on their electrophysiological phenotype (highly negative RMP, low *R*_*m*_, linear IV‐curves) and their coupling properties.

For network assessments, SRB was allowed to diffuse in whole‐cell mode. RMP and passive membrane properties were measured every 30 min to monitor cell health and access. To examine cell number and network extension over time, z‐stacks were imaged with a step size of 1 μm and a ×1–×2 digital zoom every 30 min. The ROI of the area extension of each network was drawn based on a defined threshold used for all networks and all time points using Fiji ImageJ (V1.53c). Cells were manually counted within the defined ROI of each network using the Cell Counter plugin.

To evaluate the networks' dependence on gap junctions, 100 μM carbenoxolone (CBX, Alfa Aesar J63714; three drops of anti‐foam necessary, Sigma A5757) was bath applied in an open superfusion system for >30 min before establishing a whole‐cell configuration. Network extension was imaged 15 min after going whole‐cell and the number of cells coupled was counted. Control recordings were performed for the 15 min time period and anti‐foam usage.

For real‐time gap junction coupling, data were collected from somata of SRB‐filled astrocytes in a single optical plane. Time series recordings were performed at 1 Hz. Only astrocytes directly adjacent with no intersection to the patched astrocytes were analyzed to exclude second‐degree coupling. Data are expressed as changes in fluorescence intensity relative to baseline fluorescence (Δ*F*/*F*). Raw values were used to calculate the time constant (tau). tau was calculated by measuring the monophasic, exponential arrival of SRB in coupled cells. Calculation was done in GraphPad Prism (V6.01) for every coupled cell and the mean tau values of all coupled cells for every patched astrocytes is presented. Only astrocytes with a stable RMP (i.e., <−70 mV) before and after imaging and a *R*_*s*_ < 15 MΩ were used for analysis. “Kissing” astrocytes (i.e., touching somas) were excluded from analysis.

### 2.8 Imaging of spontaneous calcium events

Induction of GCaMP6f‐expression and preparation of acute transversal spinal cord slices were as described above. A single slice was transferred to the recording chamber, fixed with a grid and allowed to acclimate for >5 min before any intervention. Continuous superfusion of oxygenated recording solution was maintained at a rate of 3.5 ml/min in an open system at all times. All recordings were performed at room temperature. Time series images were acquired at a single focal plane with a ×5 digital zoom at 0.5 Hz for 5 min. Bath application of normal recording solution (aCSF), 1 μM Tetrodotoxin (TTX; Tocris 1069) or nominally Ca^2+^‐free recording solution was carried out after 5 min of baseline recording (CTRL) using an open superfusion system and continuously maintained for >5 min before start of the next imaging frame of 5 min.

Analyses of time‐lapse image series were performed using Fiji ImageJ (V1.53c). If necessary, XY‐drift was corrected using a correction plugin (Multi DriftCorrection V1.1. by Brandon Brown). Recordings with a z‐drift were excluded from analysis. For analyzing spontaneous calcium events, ROIs were defined for astrocytic somas during baseline recording. The identical ROIs were used to analyze calcium events after bath application of normal aCSF or TTX. Spontaneous events were quantified using a threshold‐based event search in Clampfit (V10.7) without manual correction. Event rate per min per ROI was measured. The mean event rate for all somatic events per lamina was calculated per slice. Events were identified based on amplitudes that were at least twofold above the baseline standard deviation of the respective Δ*F*/*F* trace.

To assess the influence of extracellular calcium on the baseline calcium levels in astrocytes, the change in the mean intensity was calculated from 10 averaged frames before and after bath application of nominally Ca^2+^‐free recording solution. Identical ROIs were used.

### 2.9 Statistical analysis

Statistical tests were all run in Sigma Plot (V13) except for the two‐way ANOVA performed in GraphPad Prism (V6.01). Raw data points with calculated mean ± standard error of mean (SEM) are shown for all sets of experiments. All data were tested for normality using the Shapiro–Wilk normality test and the appropriate parametric or nonparametric tests were used accordingly.

For before/after comparison, a two‐tailed paired Student's *t* test or Wilcoxon signed‐rank test was used. For multiple groups, a one‐way ANOVA was used with appropriate post hoc tests for comparison between groups. The exact post hoc correction method used as well as additional corrections for multiple parameters tested are always stated in each figure legend. A two‐way ANOVA with appropriate post hoc tests was used to compare group effects over time.

Significance was set at *p* < .05, but stated in each case with a precise *p* value. In all figures significance levels are depicted as *(*p* < .05), **(*p* < .01), and ***(*p* < .001). Throughout the manuscript, the results of statistical tests (*p* values and *n*‐numbers) are reported in full in the figure legends to save space in the main body of the manuscript and to enhance clarity in the figures. *n* is defined as the number of cells or slices, *N* is defined as the number of mice. The unit of analysis is stated in each figure and figure legend. Appropriate sample sizes were based on best practices in literature, on ethical standards to minimize numbers of animals for experiments and by sample size calculations based on pilot studies. Part of these data was submitted as a diploma thesis by Felix J. Resch.

## 3 RESULTS

In this study, we compared properties of spinal astrocytes at an anatomical, molecular, electrophysiological and functional level within L1, L2, and L3 of the upper dorsal horn of the spinal cord of mice.

### 3.1 Astrocyte density and cell coverage in the upper dorsal horn

**FIGURE 1:**
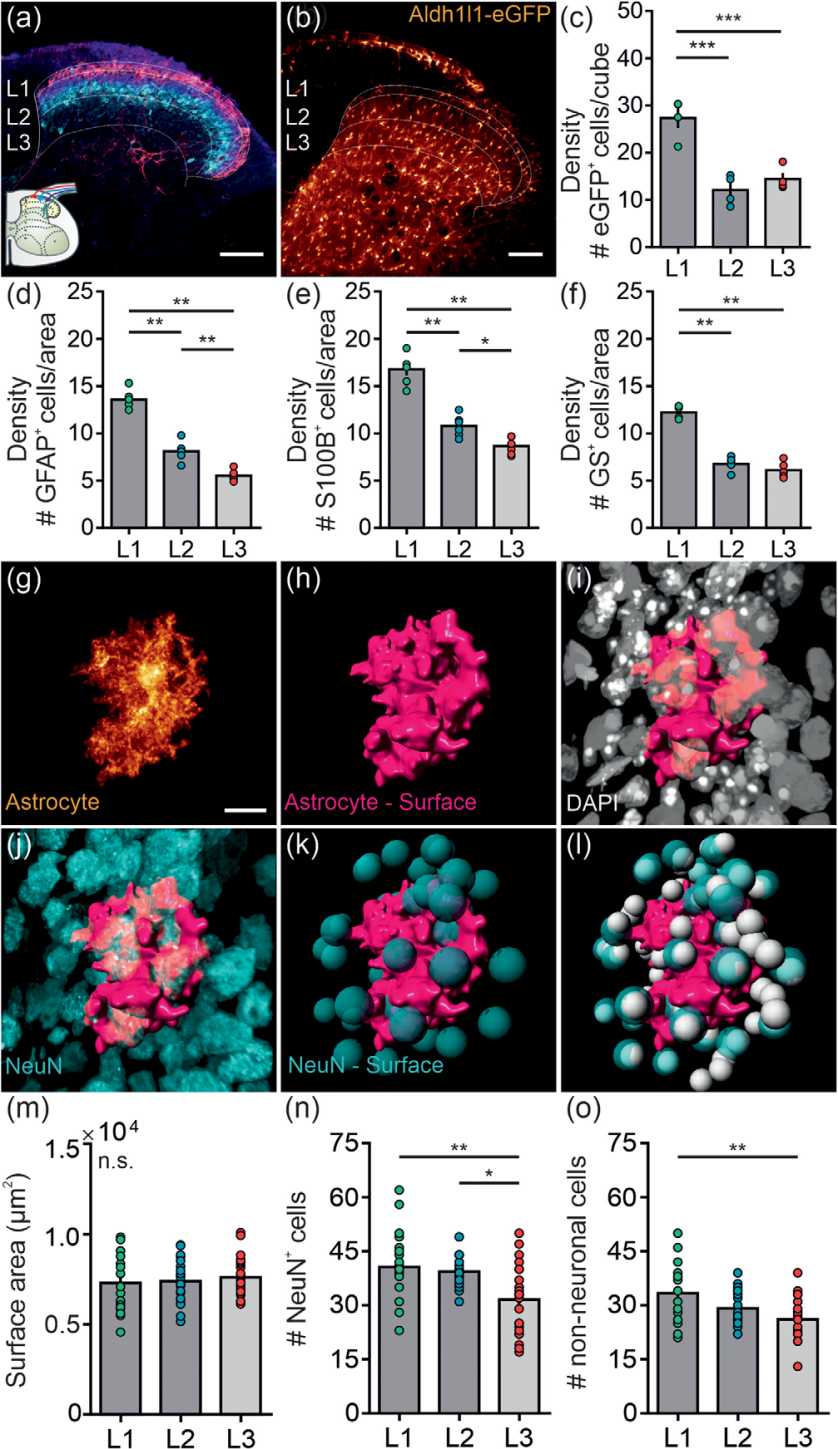
Density and anatomical properties of astrocytes in the upper dorsal horn. (a) Confocal image of the laminar structure of the upper dorsal horn. CGRP^+^‐fibers mark L1 (red), IB4^+^‐positive fibers (blue) and PKCγ‐positive interneurons (cyan) mark L2. L3 was defined using landmarks. (b) Confocal image of a cleared spinal cord section of Aldh1l1‐eGFP transgenic mice. Astrocytes are presented in glow color scheme. (c) Analysis of the astrocyte density in Aldh1l1‐eGFP transgenic mice, that is, total number of eGFP^+^ cells in a cube of 100 μm side length (*N* = 4 mice; 1 mm thick slices). L1 had a higher astrocyte density compared to L2 (27.3 ± 1.8 vs. 12.1 ± 1.4 cells; *p* < .001) and L3 (27.3 ± 1.8 vs. 14.4 ± 1.1 cells; *p* < .001). The density in L2 and L3 was similar (12.1 ± 1.4 vs. 14.4 ± 1.1 cells; *p* = .357). One‐way ANOVA with Holm–Sidak post hoc correction was performed. (d–f) Analysis of the astrocyte density in wild‐type mice, that is, total number of GFAP^+^‐(d), S100B^+^‐(e) or GS^+^ cells (f) in an area of 100 μm side length (*N* = 6 mice, mean of four slices each). One‐way ANOVA with Holm–Sidak post hoc correction was performed and further corrected for three parameters tested (density, mean intensity, %co‐localization). (d) The density of GFAP^+^ cells was significantly different across all laminae tested with the highest density in L1 (L1: 13.6 ± 0.4, L2: 8.1 ± 0.4, L3: 5.5 ± 0.2 cells; *p* < .01 for all comparisons). (e) The density of S100B^+^ cells was significantly different across all laminae tested with the highest density in L1 (L1: 16.8 ± 0.6, L2: 10.8 ± 0.4, L3: 8.7 ± 0.3 cells; L1 vs. L3 *p* < .01, L2 vs. L3 *p* < .01, L2 vs. L3 *p* = .027). (f) The density of GS^+^ cells was significantly higher in L1 compared to L2 (12.2 ± 0.2 vs. 6.8 ± 0.3 cells; *p* < .01) and L3 (12.2 ± 0.2 vs. 6.1 ± 0.3 cells; *p* < .01). The density in L2 and L3 was similar (6.8 ± 0.3 vs. 6.1 ± 0.3 cells; *p* = .345). (g) Confocal image of a single spinal astrocyte immunolabeled for GFP in Aldh1l1‐CreER^T2^ × Ai95 mice subject to a sparse‐expression protocol. (h) 3D‐surface reconstruction of the territory of the astrocyte presented in (g). (i) Immunolabeling for DAPI (grey) surrounding the astrocyte surface. (j) Immunolabeling for NeuN (cyan) surrounding the astrocyte surface. (k) 3D‐sphere representation of NeuN‐immunolabeling presented in (j) in the astrocyte surrounding only. (l) 3D‐surface reconstruction of an astrocyte with spherical representation of DAPI (grey) and NeuN (cyan) in its surrounding. (m) Analysis of the surface area of spinal astrocytes located in L1, L2, or L3 (*n* = 20–24 cells per lamina, *N* = 4 mice). The surface area of astrocytes is similar across all laminae tested (L1: 0.73 ± 0.3, L2: 0.74 ± 0.3, L3: 0.76 ± 0.2 μm^2^; *p* = ~1). (n) Analysis of the number of NeuN^+^ cells within 10 μm distance of the astrocytic surface. Astrocytes in L1 and L2 had more NeuN^+^ cells in their direct vicinity than astrocytes in L3 (40.6 ± 2.2 and 39.3 ± 0.9 vs. 31.5 ± 1.9 cells; *p* = .006 and .015). The number of neighboring NeuN^+^ cells was similar for L1 and L2 astrocytes (40.6 ± 2.2 vs. 39.3 ± 0.9 cells; *p* = ~1). (o) Analysis of the number of nonneuronal cells (DAPI^+^NeuN^−^ cells) within 10 μm distance of the astrocytic surface. Astrocytes in L1 had more nonneuronal cells in their direct vicinity than astrocytes in L3 (33.3 ± 2.0 vs. 26.1 ± 1.2 cells; *p* = .009). The number of nonneuronal cells neighboring L2 astrocytes was similar to L1 astrocytes (33.3 ± 2.0 vs. 29.1 ± 1.1 cells; *p* = .33) and L3 astrocytes (29.1 ± 1.1 vs. 26.1 ± 1.2 cells; *p* = .42). One‐way ANOVA with Holm–Sidak post hoc correction was performed and further corrected for three parameters tested (surface area, # NeuN^+^ cells, # nonneuronal cells). All data are presented as raw data with mean ± SEM. n.s. not significant, *p < .05, **p < .01, ***p < .001. Scale bar: 100 μm (a–b) and 10 μm (g–l) [Color figure can be viewed at wileyonlinelibrary.com]

Astrocytes are widely referred to as the most abundant glial cell type within the CNS (von Bartheld, Bahney, & Herculano‐Houzel, 2016). To determine if astrocytes in L1, L2, and L3 are evenly distributed across the upper dorsal horn of the spinal cord (Figure 1a), we analyzed the four prototypical astrocyte markers Aldh1l1 (Figure 1b), GFAP, S100B, and GS by using a clearing or staining approach in transgenic and wild‐type mice, respectively. Collectively with all four markers, a significantly higher astrocyte density was found in L1 compared to L2 and L3 ranging up to a 2.5‐fold difference (Figure 1c–f). A gradual decline in astrocyte density was shown for GFAP^+^‐ and S100B^+^ cells, with a significantly higher density in L1 compared to L2 and L3 and a higher density in L2 compared to L3 (Figure 1d,e).

Exploring the anatomical interplay of astrocytes with cells in their surrounding is necessary to understand their potential interactions and influences upon neighboring neuronal and nonneuronal cells. We investigated the territory of single astrocytes residing in L1, L2, or L3, to evaluate if the difference in the overall astrocytic density relates to a differential coverage of neighboring neurons and nonneuronal cells. Sparse labeling of astrocytes was achieved using a low‐dose tamoxifen protocol in Aldh1l1‐CreER^T2^ × Ai95 double transgenic mice combined with post hoc labeling for GFP, DAPI, and NeuN. In addition, IB4‐ and PKCγ‐labeling was used to assign the laminar location of the respective astrocyte. We performed 3D‐surface reconstruction of the GFP^+^‐, NeuN^+^‐, and DAPI^+^ cells (Figure 1g–l; Movie S1) to quantify the overall surface area and the number of neurons and nonneuronal cells in the astrocytic territory (Figure 1m–o). The surface area of spinal astrocytes of the upper dorsal horn was not significantly different, indicating a similar morphology and extension across all astrocytes and laminae tested (Figure 1m). We further assessed the overall territory coverage of neurons (NeuN^+^ cells) and nonneuronal cells (DAPI^+^ NeuN^−^ cells) within a distance of 10 μm of a single astrocyte residing in L1, L2, or L3. A single astrocyte within the superficial laminae L1 and L2 had on average 8.4 ± 0.4 more neurons in their direct vicinity than astrocytes within L3 (Figure 1n *)*. Additionally, a single L1 astrocyte was surrounded by 7.2 ± 0.8 more nonneuronal cells compared to L3 astrocytes with L2 astrocytes being intermediary (Figure 1o).

### 3.2 Degree of co‐localization of prototypical astrocyte markers in the upper dorsal horn

Astrocytes are classically identified by the use of a limited set of astrocyte‐specific markers. The discussion of potentially nonoverlapping populations of astrocytes is thereby often neglected. Here, we defined the percentage of overlap of the prototypical markers GFAP, S100B, and GS in spinal astrocytes and tested whether the degree of co‐localization is similar across the laminae of the upper dorsal horn.

**FIGURE 2:**
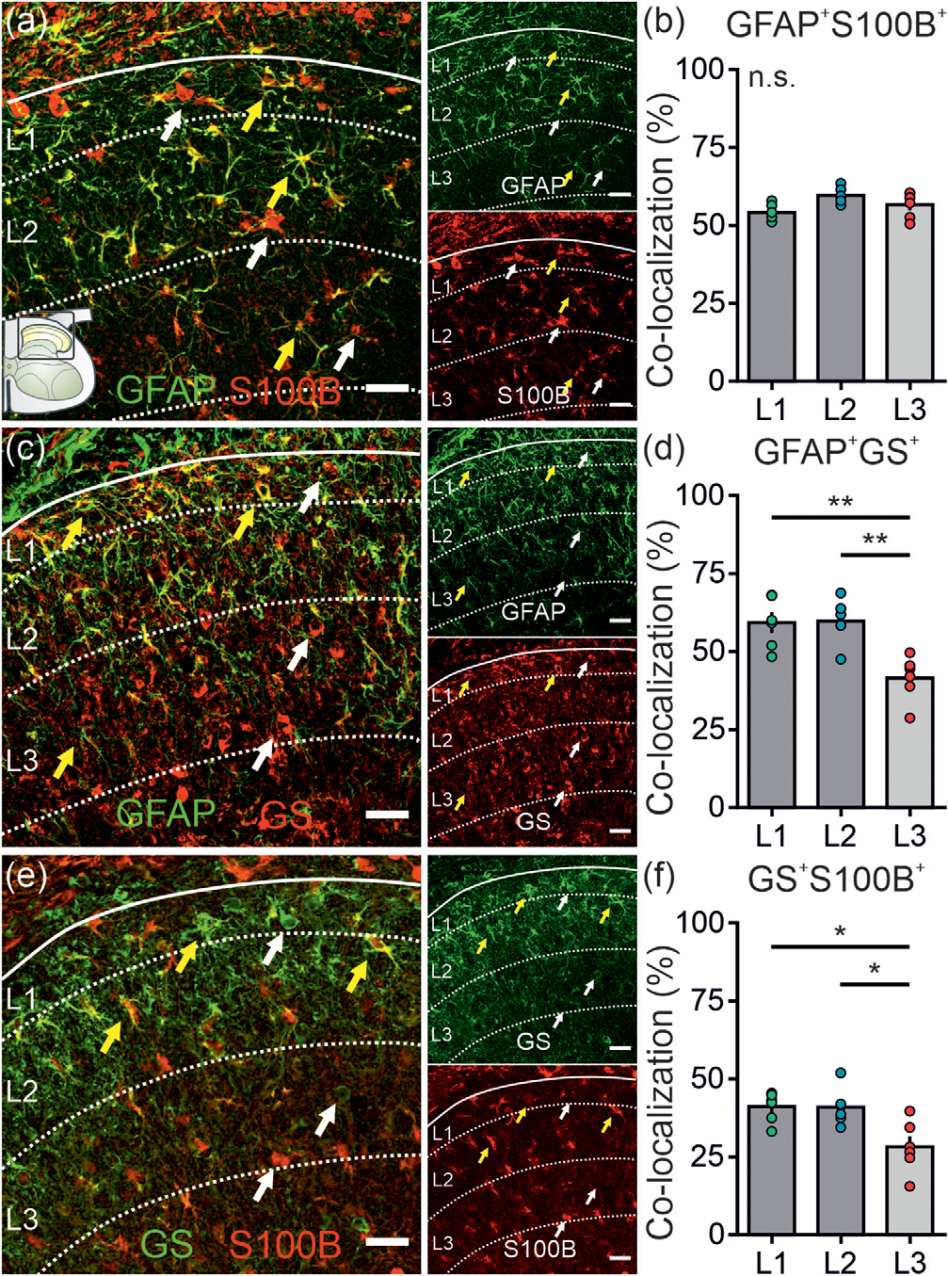
Degree of co‐localization for prototypical astrocyte markers in the upper dorsal horn. Immunolabeling combining two prototypical astrocyte markers each (GFAP, S100B and GS). IB4 and PKCγ staining served as orientation markers for L2, DAPI labeling was used to identify somatic co‐localization (for clarity not shown). Merged and single channel images are shown. Exemplary co‐localization is indicated with yellow arrows, no co‐localization with white arrows. (a) Confocal images with GFAP^+^ cells in green and S100B^+^ cells in red. (b) Analysis of the degree of co‐localization (in %) of GFAP^+^‐ and S100B^+^ cells. The degree of co‐localization was similar across all laminae (L1: 54.2 ± 1.0%, L2: 59.7 ± 1.0%, L3: 56.7 ± 1.7%; *p* = .09). (c) Confocal images with GFAP^+^ cells in green and GS^+^ cells in red. (d) Analysis of the degree of co‐localization (in %) of GFAP^+^‐ and GS^+^ cells. The degree of co‐localization was significantly higher in both L1 and L2 when compared to L3 (59.2 ± 3.3% and 59.7 ± 2.9% vs. 41.5 ± 2.9%; both *p* = .006). The degree of co‐localization was similar in L1 and L2 (59.2 ± 3.3% vs. 59.7 ± 2.9%; *p* = ~1). (e) Representative confocal images with GS^+^ cells in green and S100B^+^ cells in red. (f) Analysis of the degree of co‐localization (in %) of S100B^+^‐ and GS^+^ cells. The degree of co‐localization was significantly higher in L1 and L2 both compared to L3 (41.1 ± 1.9% and 41.0 ± 2.5% vs. 28.2 ± 3.4%; *p* = .033 and *p* = .024). The degree of co‐localization was similar in L1 and L2 (41.1 ± 1.9% vs. 41.0 ± 2.5%; *p* = ~1). *N* = 6 mice (mean of four slices/mouse). Scale bar: 20 μm. Data are presented as raw data with mean ± SEM. One‐way ANOVA with Holm–Sidak post hoc correction was performed and further corrected for 3 parameters tested (density, mean intensity, co‐localization). n.s. not significant, *p < .05, **p < .01 [Color figure can be viewed at wileyonlinelibrary.com]

We paired two astrocyte markers each with stainings for DAPI, IB4, and PKCγ to assess the somatic co‐localization in the defined laminae of the dorsal horn. Only DAPI^+^‐co‐localizations were counted. The analyses were summarized in Table 1. About 60% of all GFAP^+^‐ and S100B^+^‐cells were positive for both proteins with a similar co‐localization level across all laminae tested (Figure 2a,b). When analyzing the co‐localization of GFAP and GS, a 20% higher degree of overlap was detected in the superficial laminae L1 and L2 compared to L3. The degree of co‐localization of GFAP and GS in L1 and L2 at about 60% was comparable (Figure 2c,d). About 40% of all S100B^+^‐ and GS^+^‐cells were double positive for both proteins in L1 and L2 with a significantly lower degree of overlap in L3 with only about 30%. The degree of co‐localization in L1 and L2 was similar (Figure 2e,f).

**TABLE 1:** Summary of IHC data

Mean intensity (au)	L1	L2	L3	Co‐localization (%)	L1	L2	L3
GFAP	80.4 ± 3.9	63.8 ± 1.3	32.6 ± 1.0	GFAP^+^S100B^+^ cells	54.2 ± 1.0	59.7 ± 1.0	56.7 ± 1.7
S100B	65.3 ± 3.9	56.7 ± 2.9	64.9 ± 3.5	GFAP^+^GS^+^ cells	59.2 ± 3.3	59.7 ± 2	41.5 ± 2.9
GS	70.4 ± 7.4	75.2 ± 6.7	61.0 ± 7.0	GS^+^S100B^+^ cells	41.1 ± 1.9	41.0 ± 2.5	28.2 ± 3.4
NDRG2	62.6 ± 4.6	65.2 ± 5.5	51.6 ± 4.3	**GFAP^+^ cells**	**L1**	**L2**	**L3**
Cx43	77.8 ± 7.1	74.5 ± 2.7	50.3 ± 1.6	+ S100B^+^	60.1 ± 0.8	67.5 ± 0.8	65.0 ± 1.1
Cx30	59.6 ± 3.5	81.0 ± 3.6	71.8 ± 3.8	+ GS^+^	65.3 ± 3.8	61.8 ± 2.6	50.3 ± 3.5
GLAST	63.5 ± 5.4	74.6 ± 5.1	41.3 ± 3.0	**S100B^+^ cells**	**L1**	**L2**	**L3**
GLT1	73.5 ± 6.1	73.9 ± 6.4	59.6 ± 7.6	+ GFAP^+^	48.4 ± 1.7	51.9 ± 2.4	48.3 ± 2.9
AQP4	85.2 ± 3.9	68.6 ± 3.9	49.9 ± 2.3	+ GS^+^	33.2 ± 2.9	30.3 ± 2.1	23.0 ± 2.9
Kir4.1	75.4 ± 4.8	103.6 ± 5.6	104.0 ± 5.5	**GS^+^ cells**	**L1**	**L2**	**L3**
**Density (#cells/area)**	**L1**	**L2**	**L3**	+ GFAP^+^	52.8 ± 3.0	57.8 ± 3.5	32.8 ± 2.9
GFAP^+^ cells	14.6 ± 0.4	8.1 ± 0.4	5.5 ± 0.2	+ S100B^+^	48.8 ± 1.3	51.2 ± 2.9	33.0 ± 4.0
S100B^+^ cells	16.8 ± 0.6	10.8 ± 0.5	8.7 ± 0.4	**Density (#cells/cube)**	**L1**	**L2**	**L3**
GS^+^ cells	12.2 ± 0.3	6.8 ± 0.3	6.1 ± 0.3	eGFP^+^ cells	27.3 ± 1.8	12.1 ± 1.4	14.4 ± 1.1

*Note*: Table summarizing the analyses from all IHC stainings referring to Figures 1, 2, 3. Statistical significances are not indicated. Data are presented as mean ± SEM.

### 3.3 Expression profile of astrocyte‐enriched proteins in the upper dorsal horn

Astrocytes are equipped with a plethora of receptors, channels, transporters and other molecules enabling them to fulfil multitude of tasks. A molecular characterization of astrocyte‐enriched proteins allowed comparison between astrocytes of the distinct laminae of the upper dorsal horn, and therefore, the identification of lamina‐specific differences between them.

**FIGURE 3:**
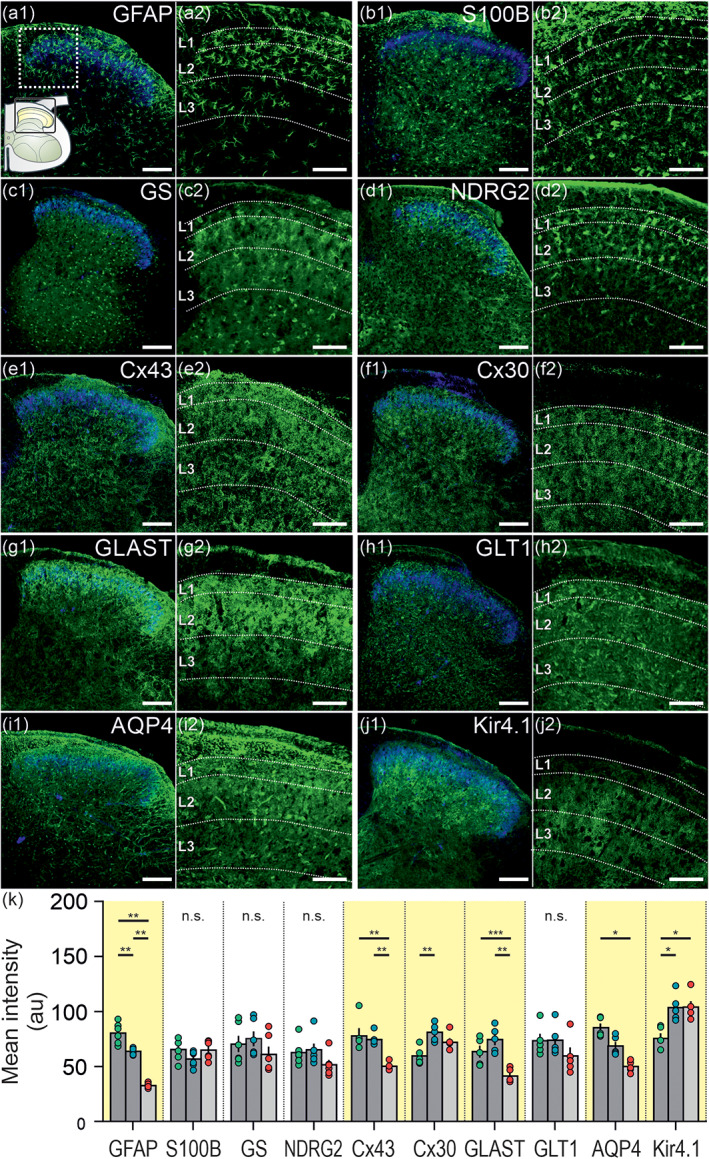
Expression profile of astrocyte‐enriched proteins in the upper dorsal horn. Immunolabeling for 10 astrocyte‐enriched proteins (green). IB4 and PKCγ staining (both in blue) served as orientation markers for L2. Confocal images of the spinal cord dorsal horn (1) and the respective magnification of L1, L2, and L3 (2) are shown for the following proteins: GFAP (a1, a2), S100B (b1, b2), GS (c1, c2), NDRG2 (d1, d2), Cx43 (e1, e2), Cx30 (f1, f2), GLAST (g1, g2), GLT1 (h1, h2), AQP4 (i1, i2), and Kir4.1 (j1, j2). Scale bar: 100 μm (1) and 50 μm (2). (k) Analysis of the mean intensity (au) in L1, L2, and L3. *N* = 5–6 mice (mean of 4–6 slices/mouse). GFAP (*p* < .01), S100B (*p* = .522), GS (*p* = .543), NDRG2 (*p* = .073), Cx43 (*p* = .009), Cx30 (*p* = .004), GLAST (*p* < .001), GLT1 (*p* = 0.114), AQP4 (*p* = .003), and Kir4.1 (*p* = .009). Data are presented as raw data with mean ± SEM. One‐way ANOVA with Holm–Sidak post hoc correction was performed. Values for GFAP, S100B and GS were further corrected for three parameters tested (density, mean intensity, co‐localization). In case normality failed, a Kruskal–Wallis one‐way ANOVA with Tukey post hoc correction was performed (GS, NDRG2, Cx43, GLT1, AQP4, and Kir4.1). n.s. not significant, *p < .05, **p < .01, ***p < .001 [Color figure can be viewed at wileyonlinelibrary.com]

We paired the staining for each astrocyte‐enriched protein with stainings for DAPI, IB4 and PKCγ to define the laminar‐specific ROIs. To evaluate lamina‐ and circuit‐specific differences, we compared the mean intensity of each protein of interest between L1, L2, and L3. Proteins of interest were reflecting key functions of astrocytes: the prototypical markers GFAP, S100B, GS, and NDRG2, the two connexins important for gap junction coupling: Cx43 and Cx30, two prominent glutamate transporters: GLAST (EAAT1) and GLT1 (EAAT2), the water channel AQP4, and the inwardly rectifying K^+^‐channel Kir4.1. All data are shown in Figure 3k and summarized in Table 1.

Apart from the prototypical astrocyte markers S100B (Figure 3b), GS (Figure 3c) and NDRG2 (Figure 3d) as well as GLT1 (Figure 3h), differences in the expression profile across the laminae of the upper dorsal horn were found for all other proteins tested indicating substantial circuit specificity. GFAP (Figure 3a), Cx43 (Figure 3e), GLAST (Figure 3g), and AQP4 (Figure 3i) were enriched in the nociceptive laminae L2 and/or L1 compared to L3. Whereas, Cx30 (Figure 3*f*) and Kir4.1 (Figure 3j) were significantly lower in L1 compared to L2 and/or L3.

### 3.4 Properties of astrocytic networks and real‐time gap junction coupling of astrocytes in the upper dorsal horn

Astrocytes are interconnected via gap junctions allowing them to work as a syncytium (Dermietzel, Hertberg, Kessler, & Spray, 1991). We found marked differences in the expression profile of the prototypical gap junction protein Cx43 indicating a lamina‐ and circuit‐specific difference in the coupling properties of spinal astrocytes. We therefore analyzed the network extension, the inherent dependence on gap junctions per se and the real‐time coupling of astrocytes residing in L1, L2, or L3 of the upper dorsal horn to assess if the properties and degree of coupling were similar across lamina borders.

To do so, we performed whole‐cell patch‐clamp recordings of a single astrocyte residing in a defined lamina with the low molecular weight dye SRB included in the patch pipette. Diffusion of the dye was monitored over prolonged period of times (i.e., every 30 min for 120 min) or in real‐time.

**FIGURE 4:**
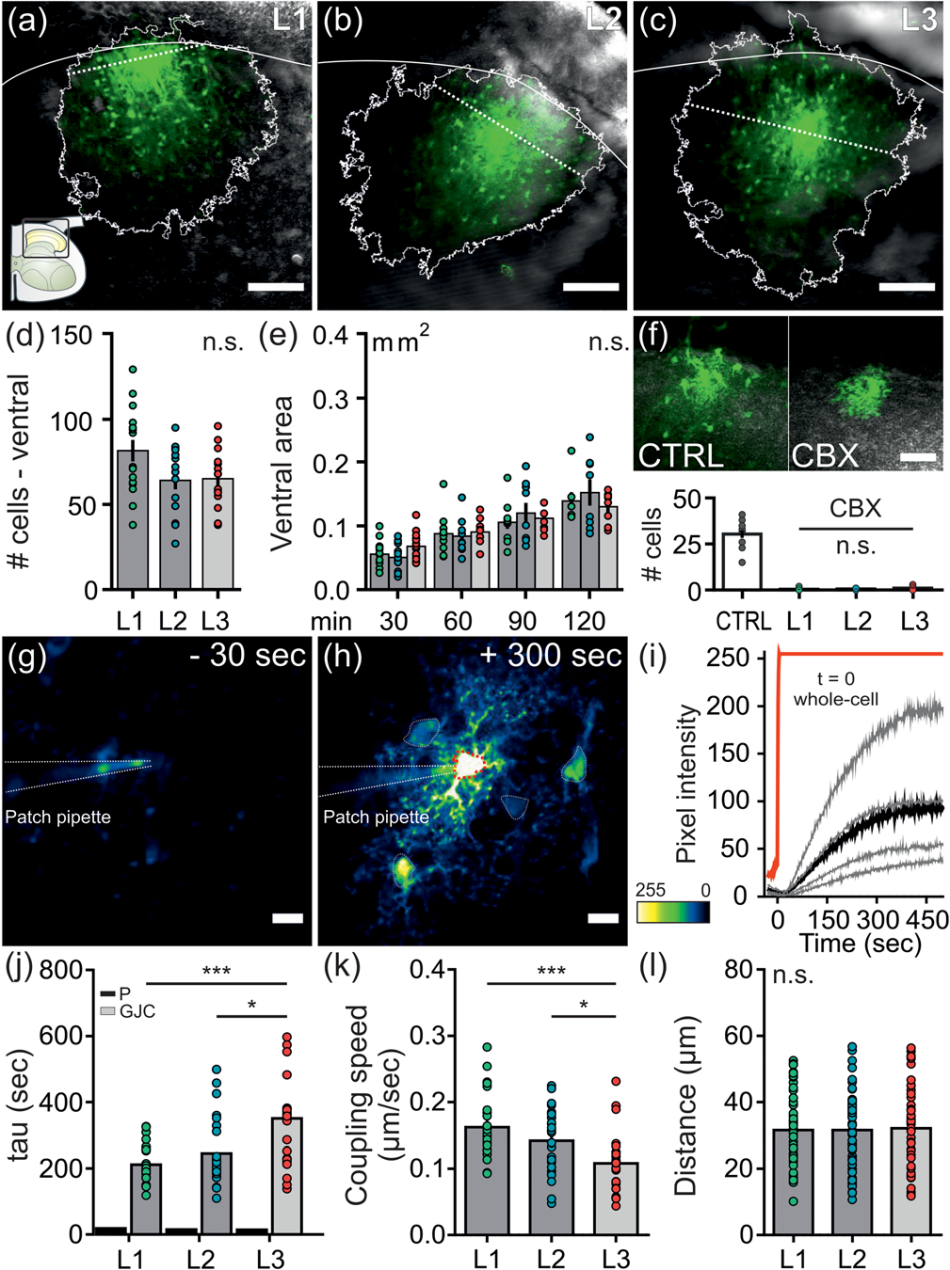
Properties of astrocytic networks and real‐time gap junction coupling in the upper dorsal horn. Whole‐cell patch‐clamp recordings were performed from astrocytes located in L1, L2, or L3 with the low molecular weight dye SRB (0.1 mg/ml) included in the patch pipette. Diffusion of SRB through astrocytic gap junctions was monitored over time, in real‐time or in the presence of a gap junction blocker. (a–c) Representative images of z‐stack projections of astrocytic networks from astrocytes patched in L1 (a), L2 (b), or L3 (c). Thresholded area is outlined in white. Dotted line represents the dorsal‐ventral partition. Scale bar: 100 μm. (d) Cell count of coupled astrocytes in the ventral region of a single patched cell after 30 min of SRB diffusion. The number of coupled cells ventral to the patched cell was similar across all laminae (L1: 81.6 ± 6.3, L2: 64.1 ± 5.2, L3: 65.1 ± 4.4 cells; *p* = .09). *n* = 15–16 patched cells per lamina. *N* = 31 mice. One‐way ANOVA with Holm–Sidak post hoc correction was performed and further corrected for two parameters tested (cell count, ventral area). (e) Analysis of the ventral area of networks from astrocytes patched in L1, L2, or L3 over time. The ventral area of all networks was similar over time (30–120 min; L1: 0.06 ± 0.00–0.14 ± 0.01 mm^2^, L2: 0.05 ± 0.00–0.15 ± 0.02 mm^2^, L3: 0.07 ± 0.01–0.13 ± 0.01 mm^2^). *n* = 15–16 cells per lamina at 30 min; *N* = 31 mice. Two‐way ANOVA with Tukey post hoc correction was performed and further corrected for 2 parameters tested (cell count, ventral area; time: *p* < .001, lamina: *p* = ~1, interaction: *p* = .933). (f) Images of z‐stack projections after 15 min of coupling under control conditions (CTRL) and in the presence of the gap junction blocker CBX (100 μM for >30 min). Under CBX treatment, network formation in L1, L2, and L3 astrocytes was prevented to a similar extent (*p* = .231; *n* = 12 CTRL, *n* = 9–11 cells per lamina for CBX treatment, *N* = 6 mice). Kruskal–Wallis one‐way ANOVA was performed. (g–h) Images of real‐time astrocytic gap junction coupling before (g) and after (h) establishing whole‐cell configuration. (i) Representative traces showing the kinetics of SRB filling of the patched cell (red trace) and kinetics of the coupling of neighboring cells (grey traces; mean is presented in black). The increase in somatic pixel intensity is plotted against time (s). (j) tau values of the coupling of astrocytes patched in L1, L2, or L3. tau values for patched cells (P; black bars; L1: 18.5 ± 4.6 s, L2: 15.4 ± 3.8 s, L3: 14.5 ± 3.5 s) and mean of coupled cells for each patched cell (GJC; grey bars) are shown. Coupled astrocytes in L1 and L2 had significantly smaller tau values compared to coupled astrocytes in L3 (211.5 ± 11.7 s and 245.5 ± 20.5 s vs. 350.3 ± 37.2 s; *p* < .001 and *p* = .01). tau values of coupled astrocytes in L1 and L2 were similar (211.5 ± 11.7 s and 245.5 ± 20.5 s; *p* = .348). (k) tau value correction for inter‐astrocytic distance is given as coupling speed (μm/s). Coupled astrocytes in L1 and L2 had a significantly faster coupling speed compared to coupled astrocytes in L3 (0.16 ± 0.01 μm/s and 0.14 ± 0.01 s vs. 0.11 ± 0.01 s; *p* < .001 and *p* = .03). (l) Total inter‐astrocytic distances of soma center points of coupled cells to patched cell are similar between laminae (L1: 31.5 ± 1.4 μm, L2: 31.5 ± 1.6 μm, L3: 32.1 ± 1.7 μm; *p* = .973). *n* = 25 patched cells per lamina, total of 52–59 coupled cells per lamina, *N* = 18 mice. Data are presented as raw data with mean ± SEM. Kruskal–Wallis one‐way ANOVA with Tukey post hoc correction was performed. n.s. not significant, *p < .05, **p < .01, ***p < .001 [Color figure can be viewed at wileyonlinelibrary.com]

These experiments revealed that spinal astrocytes of L1, L2, and L3 form extensive networks across lamina borders (Figure 4a–c). The natural dorsal border of the dorsal horn limits dye spread for astrocytes in L1 and also L2. We thus only analyzed the free diffusion ventral to the patched astrocytes. After 30 min of diffusion, 70 ± 5 cells were coupled on average. The number of coupled SRB^+^ cells was similar for astrocytes patched in L1, L2, or L3 (Figure 4d). We further monitored the area extension of the astrocytic networks every 30 min for 2 hr to investigate the network formation over time in more detail. Also over time, the degree of coupling was similar between spinal astrocytes patched in L1, L2, and L3 of the dorsal horn (Figure 4e). Incubating the spinal slices for >30 min with the nonselective gap junction blocker CBX completely prevented any dye spread to neighboring cells in all three laminae tested (Figure 4f).

We then monitored the coupling of spinal astrocytes in real‐time. After establishing a whole‐cell configuration with the patched astrocyte (Figure 4g,h; Movie S2), the mean time constant value (tau, in seconds) was calculated for the monophasic, exponential arrival of SRB in directly neighboring coupled cells (Figure 4i). Coupled astrocytes in the superficial laminae L1 and L2 displayed significantly lower tau values compared to coupled astrocytes in L3. Whereas the average tau for coupled astrocytes in L1 and L2 was 229 ± 16 s, the average tau value for coupled astrocytes in L3 was significantly larger (350 ± 37 s; Figure 4j). Correction for the individual inter‐astrocytic distance resulted in a significantly faster coupling speed for astrocytes in L1 and L2 with on average 0.15 ± 0.01 μm/s compared to L3 astrocytes with 0.11 ± 0.01 μm/s (Figure 4k). The inter‐astrocytic distance in the analyzed focal planes with 31.7 ± 1.5 μm was similar (Figure 4l).

### 3.5 Electrophysiological properties of astrocytes in the upper dorsal horn

**FIGURE 5:**
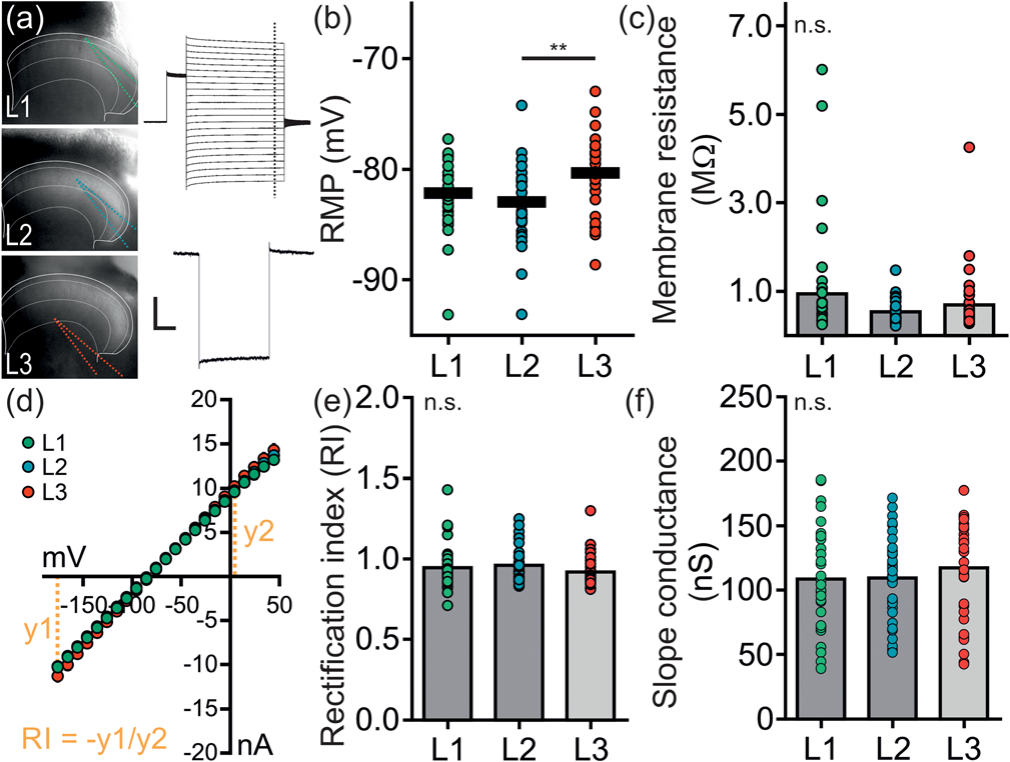
Passive membrane properties of astrocytes in the upper dorsal horn. Whole‐cell patch‐clamp recordings from spinal astrocytes were performed in L1, L2, or L3 of the spinal dorsal horn. All recordings were performed directly after establishing whole‐cell configuration. (a) Representative images of the recording sites in the dorsal horn. Original traces of an IV‐curve and membrane test of a representative astrocyte. Scale bar: 10 nA, 100 ms and 200 pA, 20 ms, respectively. (b) The RMP (mV) of spinal astrocytes located in L1, L2, or L3 was recorded at *I* = 0 (L1: −82.1 ± 0.5 mV, L2: −82.9 ± 0.5 mV, L3: −80.4 ± 0.6 mV). L3 astrocytes had a slightly more depolarized RMP compared to L2 astrocytes (*p* = .008). The RMP of L1 astrocytes was similar to the RMP of L2 and L3 astrocytes (*p* = ~1 and *p* = 0.168). (c) The *R*_*m*_ was calculated from each membrane test (*R*_*m*_ = *R*_total_ − *R*_*s*_). The *R*_*m*_ (MΩ) of spinal astrocytes was similar (L1: 0.9 ± 0.2 MΩ, L2: 0.5 ± 0.0 MΩ, L2: 0.7 ± 0.1 MΩ; *p* = ~1). (d) Plotted mean of IV‐curves from astrocytes located in L1, L2, or L3. Calculation of the RI is indicated in yellow. (e) Analysis of the RI of spinal astrocytes. The RI of spinal astrocytes was similar (L1: 0.9 ± 0.0, L2: 1.0 ± 0.0, L3: 0.9 ± 0.0; *p* = .352). (f) Comparison of the slope conductance (nS) of spinal astrocytes. The slope conductance of spinal astrocytes was similar (L1: 108.7 ± 6.2 nS, L2: 109.5 ± 5.2 nS, L3: 117.5 ± 7.1 nS). *n* = 32–39 cells per lamina, *N* = 51 mice. Data are presented as raw data with mean ± SEM. Kruskal–Wallis One‐way ANOVA with Dunn's post hoc correction was performed and further corrected for four parameters (RMP, *R*_*m*_, RI, slope conductance). n.s. not significant, **p < .01 [Color figure can be viewed at wileyonlinelibrary.com]

Astrocytes are characterized by a highly negative RMP, a low *R*_*m*_, and a linear IV‐relationship (Walz & MacVicar, 1988; Zhong et al., 2016; Zhou, Schools, & Kimelberg, 2006). We performed whole‐cell patch‐clamp recordings of single astrocytes residing in L1, L2, or L3 to investigate if spinal astrocytes of the upper dorsal horn all share these common characteristics (Figure 5a).

The electrophysiological properties of astrocytes were comparable across laminae with the exception of the RMP. Astrocytes in L3 had significantly more depolarized RMPs compared to L2 astrocytes (Figure 5b). *R*_*m*_, RI, and slope conductance were similar (Figure 5c–f).

### 3.6 The effect of Kir4.1 blockade on the electrophysiologcal properties of spinal astrocytes in the upper dorsal horn

At the synaptic level, astrocytes maintain ion homeostasis by buffering the excess of K^+^ ions after neuronal activity. Several mechanisms ranging from net uptake to energy‐efficient spatial buffering or siphoning are described to underlie this astrocyte‐specific feature (for review see Walz, 2000). The inwardly rectifying potassium channel Kir4.1 is highly expressed in astrocytes and widely considered as one of the main channels for spatial buffering (Olsen, Higashimori, Campbell, Hablitz, & Sontheimer, 2006 or for review see Nwaobi, Cuddapah, Patterson, Randolph, & Olsen, 2016; Olsen & Sontheimer, 2008). Given the differential expression of Kir4.1 across the upper dorsal horn (Figure 3j), we set out to investigate the effect of Kir4.1 blockade on the electrophysiologcal properties of spinal astrocytes residing in L1, L2, and L3.

We performed whole‐cell patch‐clamp recordings of single astrocytes under control conditions and after bath application of 100 μM BA to block Kir4.1 channels. BA is proposed to strongly impair the spatial relocation of K^+^ ions by blocking the channel necessary for passive uptake (Méndez‐González et al., 2016). We analyzed the RMP, *R*_*m*_, and slope conductance after bath application of BA and compared the relative effect of Kir4.1 blockade between laminae. Control recordings with standard aCSF were performed to exclude any time‐related effects.

**FIGURE 6:**
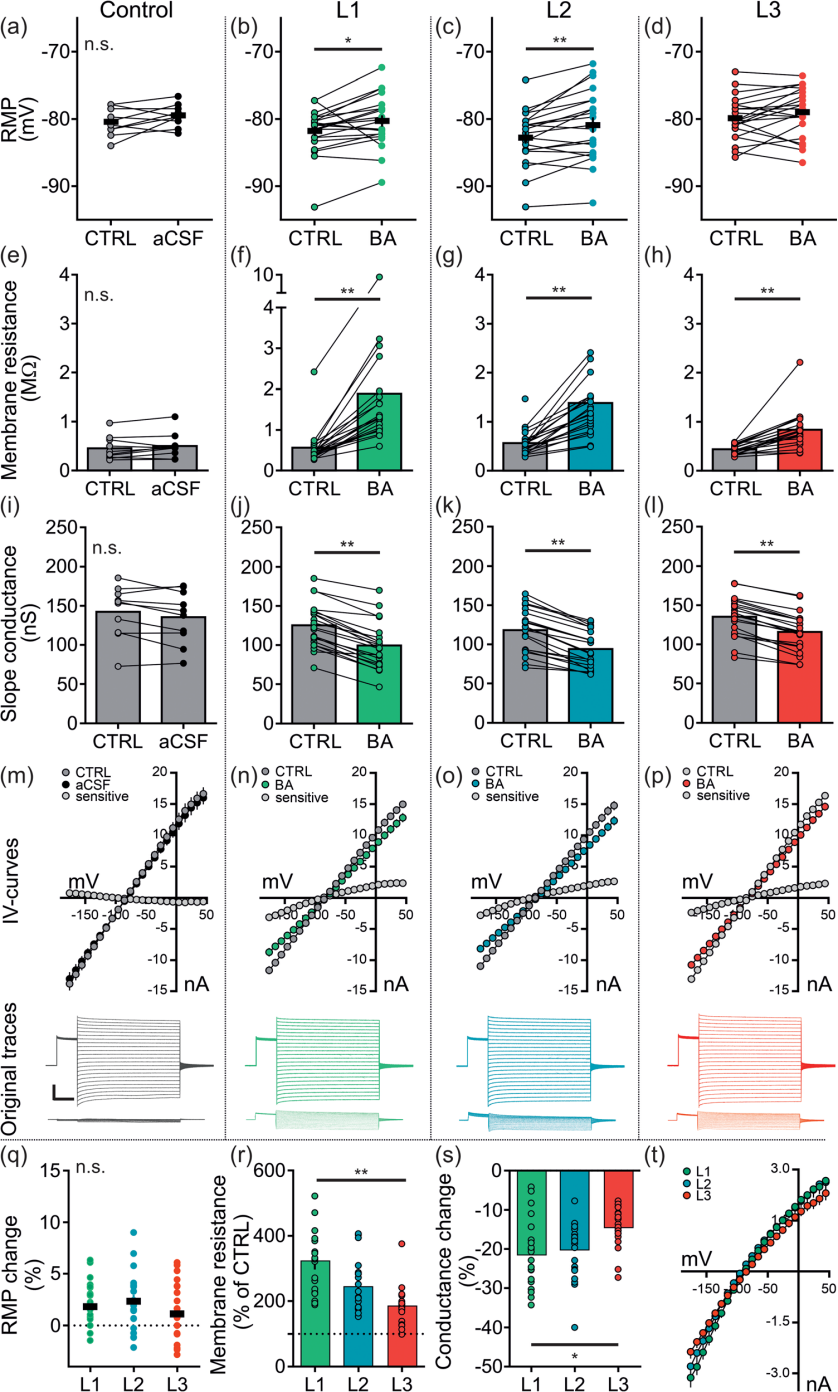
Effect of Kir4.1‐blockade on passive membrane properties of astrocytes in the upper dorsal horn. Whole‐cell patch‐clamp recordings were performed from spinal astrocytes located in L1, L2, or L3. Passive membrane properties were recorded under control conditions (CTRL) and in the presence of BA (100 μM; >5 min bath application) or standard aCSF (Control). Values for RMP, *R*_*m*_, calculated slope conductance, mean of IV‐curves and original traces for IV‐curves and subtraction currents are shown. (a–d) Recordings of the RMP (mV). (a) The RMP was stable over time (−80.4 ± 0.6 mV vs. −79.5 ± 0.5 mV; *p* = .444), BA application depolarized the RMP in (b) L1 astrocytes (−81.8 ± 0.7 mV vs. −80.3 ± 0.8 mV; *p* = .012) and (c) L2 astrocytes (82.8 ± 0.9 mV vs. −80.9 ± 1.1 mV; p < .01) with no effect on (d) L3 astrocytes (−79.9 ± 0.7 mV vs. −79.0 ± 0.8 mV; *p* = .345). (e–h) Recordings of the *R*_*m*_ (MΩ). (e) The *R*_*m*_ was stable over time (0.5 ± 0.1 MΩ vs. 0.5 ± 0.1 MΩ; *p* = .600). BA application increased the *R*_*m*_ in (f) L1 astrocytes (0.6 ± 0.1 MΩ vs. 1.9 ± 0.4 MΩ; *p* < .01), (g) L2 astrocytes (0.6 ± 0.1 MΩ vs. 1.4 ± 0.2 MΩ; *p* < .01) and (h) L3 astrocytes (0.4 ± 0.0 MΩ vs. 0.8 ± 0.1 MΩ; *p* < .01). (i–p) Calculated slope conductance (nS). (i, m) The slope conductance was stable over time (142.4 ± 10.6 vs. 135.6 ± 10.7 nS; *p* = .240). BA application reduced the slope conductance in (j, n) L1 astrocytes (125.2 ± 6.2 vs. 99.4 ± 6.5; *p* < .01), (k, o) L2 astrocytes (118.0 ± 6.2 vs. 93.9 ± 5.2; *p* < .01) and (l, p) L3 astrocytes (135.1 ± 5.6 vs. 115.8 ± 5.4; *p* < .01). (a–p) A paired *t* test or Wilcoxon signed rank test was performed and further corrected for three parameters (RMP, *R*_*m*_, slope conductance). (q–t) Comparison of the normalized change (expressed as % of CTRL) in response to bath application of BA. (q) The effect of BA application on the RMP was similar (L1: +1.8 ± 0.8%, L2: +2.3 ± 0.7%, L3: +1.0 ± 0.7%; *p* = ~1). (r) BA had a stronger effect on the *R*_*m*_ of L1 astrocytes compared to L3 astrocytes (334.8 ± 27.9% vs. 190.0 ± 13.2%; *p* < .01). The effect on L2 astrocytes was not significantly different from L1 (250.4 ± 18.8% vs. 334.8 ± 27.9%; *p* = .138) and L3 astrocytes (250.4 ± 18.8% vs. 190.0 ± 13.2%; *p* = .228). (s–t) BA had a stronger effect on the slope conductance of L1 astrocytes compared to L3 astrocytes (−22.8 ± 1.9% vs. −14.8 ± 1.2%; *p* = .027). The effect on L2 astrocytes was not significantly different from L1 (−20.6 ± 1.8% vs. −22.8 ± 1.9%; *p* = ~1) and L3 astrocytes (−20.6 ± 1.8% vs. −14.8 ± 1.2%; *p* = .081). A one‐way ANOVA with Holm–Sidak post hoc correction or Kruskal–Wallis one‐way ANOVA on ranks with Tukey post hoc correction was performed and further corrected for three parameters (RMP, *R*_*m*_, slope conductance). Control: *n* = 10 cells, *N* = 6 mice; BA: *n* = 21 cells per lamina, *N* = 15–18 mice. Data are presented as raw data with mean ± SEM. Scale bar: 5 nA, 100 ms. n.s. not significant, *p < .05, **p < .01 [Color figure can be viewed at wileyonlinelibrary.com]

We found that the RMP, the *R*_*m*_ and slope conductance were stable over time (Figure 6a,e,i,m). Bath application of BA significantly depolarized the RMP in astrocytes of laminae L1 and L2 with no effect on the RMP of L3 astrocytes (Figure 6b–d). It further induced a significant increase of the *R*_*m*_ (Figure 6f–h) as well as a significant reduction in slope conductance in L1, L2, and L3 astrocytes (Figure 6j–l, n–p). When comparing the relative effect of Kir4.1 blockade on the membrane properties of spinal astrocytes, the change in RMP with a depolarization of 1.7 ± 0.7% was similar across astrocytes (Figure 6q) whereas the effect on the *R*_*m*_ and slope conductance was differential. The increase in *R*_*m*_ by 335 ± 28% after Kir4.1 blockade was about 45% higher in L1 astrocytes as compared to astrocytes in L3, with the effect on L2 astrocytes being intermediary (Figure 6r). Similar, the reduction in slope conductance of 23 ± 2% was significantly stronger in L1 astrocytes compared to L3 astrocytes with an average reduction of 15 ± 1%. L2 astrocytes were again intermediary (Figure 6s,t).

### 3.7 The nature of spontaneous calcium signals of spinal astrocytes in the upper dorsal horn

Although astrocytes display a passive electrical profile, they are very active cells in terms of intracellular calcium signaling. Responding to a wide range of neurotransmitters and other substances, astrocytes also show vivid spontaneous fluctuations in their intracellular calcium levels (Khakh & McCarthy, 2015; Wang, Zhou, Tang, Wang, & Chai, 2006). To understand if the spontaneous calcium signals vary depending on the neuronal environment the astrocytes were residing in, the nature of the somatic calcium events of spinal astrocytes in the dorsal horn was compared between L1, L2, and L3.

**FIGURE 7:**
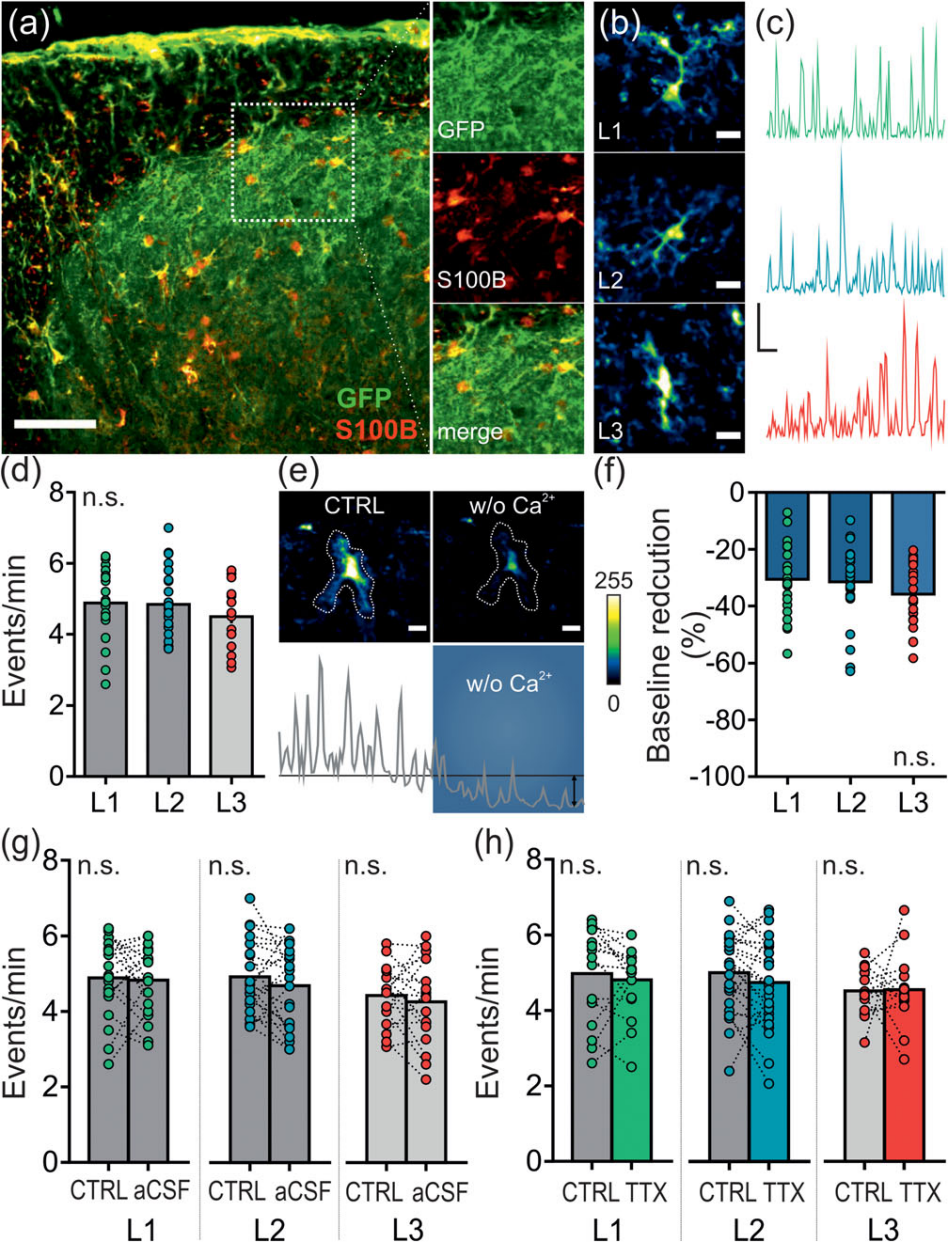
Nature of spontaneous somatic calcium signals of astrocytes in the upper dorsal horn. Astrocytic expression of GCaMP6f was induced by Tamoxifen treatment (×5 75 mg/kg; i.p.) in Aldh1l1‐CreER^T2^ × Ai95 mice. Calcium signals were monitored for 5 min before (CTRL) and after 5 min of bath application of nominally Ca^2+^‐free aCSF, normal aCSF or TTX (1 μM). (a) Immunolabeling for GFP and S100B. Images of z‐projections are shown. Scale bar: 100 μm. (b) GCaMP6f‐expressing astrocytes located in L1, L2, and L3. Scale bar: 10 μm. (c) Original traces of the somatic calcium signal of the astrocytes shown in (b). Scale bar: 0.5 Δ*F*/*F*, 30 s (d) Analysis of a 5 min time frame of somatic calcium events given as events/min in astrocytes located in L1, L2, or L3. The event rate was similar across all laminae tested (L1: 4.9 ± 0.2, L2: 4.8 ± 0.2, L3: 4.5 ± 0.2 events/min; *p* = .632). The mean event rate of all astrocytes within a lamina per slice is presented. *n* = 19–25 slices per lamina (total of 45–63 astrocytes per lamina), *N* = 12 mice. One‐way ANOVA was performed and further corrected for two parameters tested (control, over time). (e) Images and original traces of astrocytic calcium signals before and after application of nominally Ca^2+^‐free aCSF (w/o Ca^2+^; blue box). (f) Quantification of the baseline calcium signals given as normalized mean intensity change (% of CTRL). The reduction in baseline calcium was similar across all laminae tested (L1: −30.6 ± 2.5%, L2: −31.5 ± 2.4%, L3: −35.8 ± 2.5%; *p* = .327). The mean reduction of all astrocytes within a lamina per slice is presented. *n* = 20–28 slices (total mean of 49–72 astrocytes per lamina), *N* = 10 mice. One‐way ANOVA was performed. (g) Analysis of 5 min time frames of somatic calcium events given as events/min in astrocytes located in L1, L2, or L3 during sustained application of aCSF. The calcium event rate was stable over time in astrocytes located in L1 (4.9 ± 0.2 vs. 4.8 ± 0.2 events/min; *p* = ~1), L2 (4.9 ± 0.2 vs. 4.7 ± 0.2 events/min; *p* = .502) and L3 (4.4 ± 0.2 vs. 4.3 ± 0.3 events/min; *p* = .962). *n* = 18–22 slices, *N* = 12 mice. (j–l) Analysis of 5 min time frames of somatic calcium events given as events/min in astrocytes located in L1, L2, or L3 before (CTRL) and after 5 min bath application of TTX (1 μM). Blocking neuronal action potential firing had no significant effect on the somatic calcium event rate in astrocytes located in L1 (5.0 ± 0.3 vs. 4.8 ± 0.2 events/min; *p* = .448), L2 (5.0 ± 0.2 vs. 4.7 ± 0.2 events/min; *p* = .153), and L3 (4.5 ± 0.2 vs. 4.5 ± 0.2 events/min; *p* = .978). *n* = 15–27 slices, *N* = 11 mice. A paired *t* test was performed. Data are presented as raw data with mean ± SEM. n.s. not significant [Color figure can be viewed at wileyonlinelibrary.com]

We monitored spontaneous calcium signals in acute transversal spinal cord slices from Aldh1l1‐CreER^T2^ × Ai95 mice where astrocytes selectively expressed the genetically encoded calcium indicator GCaMP6f (Figure 7a–c; Movie S3; Srinivasan et al., 2016). The event rate of spontaneous calcium signals was on average 4.7 ± 0.2 events/min and was similar across all astrocytes in L1, L2, and L3 of the upper dorsal horn (Figure 7d). To address the contribution of extracellular calcium to the baseline calcium levels in spinal astrocytes, nominally Ca^2+^‐free recording solution was bath applied and the difference in the mean baseline level was assessed. Omitting extracellular calcium reduced the baseline calcium levels by 33 ± 3% (Figure 7e; Movie S3). The level of reduction was comparable in astrocytes across all laminae tested (Figure 7f). The event rate of spontaneous calcium signals was stable over time across all laminae tested (Figure 7g). Neurotransmitter released from neurons can influence the calcium levels in astrocytes (Pasti, Volterra, Pozzan, & Carmignoto, 1997; Porter & McCarthy, 1996). To investigate if neuronal activity contributes to the spontaneous calcium event rate in astrocytes, action potential firing was blocked by bath application of TTX, a blocker of voltage‐gated Na^+^‐channels. Blockade of action potential firing had no impact on the astrocytic calcium event rate across all laminae tested (Figure 7h).

## 4 DISCUSSION

Astrocytes residing in the upper dorsal horn showed distinct lamina‐ and circuit‐specific adaptations indicating a fine‐tuning of astrocytic features and properties for the processing of distinct types of sensory information in mice. Astrocytes in the superficial laminae L1 and L2 of the spinal dorsal horn were characterized by higher expression levels of GFAP, Cx43, GLAST, a better coupling strength and a higher overlap of glutamine synthetase with GFAP and S100B compared to astrocytes in L3. In addition, L1 was characterized by higher expression levels of AQP4 and astrocytes in L1 were more responsive to Kir4.1 blockade compared to astrocytes in L3. Astrocytes of the upper dorsal horn, however, shared important principle features enabling them to carry out their basic operations. Across L1‐L3, spinal astrocytes had common passive membrane properties, were coupled across lamina borders sharing overall network qualities and displayed spontaneous somatic calcium signals of similar character.

Together, these results indicate that astrocytes in L1 and L2 are better equipped for a faster and more efficient clearance and recycling of neurotransmitters and ions than astrocytes located in L3. Processing of nociceptive information in L1 and L2 is correlated with higher levels of extracellular glutamate and K^+^ ions (Al‐Ghoul et al., 1993; Heinemann et al., 1990; Kangrga & Randic, 1991). Astrocytes in the superficial laminae are well equipped to safeguard the neuronal environment from excitotoxicity after processing of nociceptive information; more proficiently than astrocytes in L3 processing tactile information. Since astrocytes execute these versatile tasks, malfunction of astrocytes has dire consequences for nociceptive signaling and, not surprisingly, astrocytes are hence strongly implicated in the pathology of chronic pain conditions (Kohro et al., 2020; or for review see Xanthos & Sandkühler, 2014; Ji, Donnelly, & Nedergaard, 2019; Li, Chen, Zhang, Zhang, & Yao, 2019).

Here, we found that L1 had an up to 2.5‐fold higher astrocyte density compared to L2 and L3. We confirmed this finding using four different prototypical astrocyte markers and two different methods. L1 holds the majority of projection neurons for supraspinal processing and is targeted mainly by peptidergic C‐fibers. Astrocytes are equipped for directly responding to neuropeptides via the respective receptors (Burmeister et al., 2017; Hansen, Vacca, Pitcher, Clark, & Malcangio, 2016; Li et al., 2015; Porter & McCarthy, 1997). To date, the role of astrocytes in the recycling and inactivation of neuropeptides is not understood. Neurotransmission with peptides such as CGRP or substance P enhance the release of glutamate and aspartate (Kangrga & Randic, 1990), thus eventually demanding more astrocytic assistance. The higher astrocytic density in L1 was also in line with the higher coverage of nonneuronal cells by L1 astrocytes observed in this study. We further found that astrocytes in L1 and L2 covered more neuronal somata on average. This may reflect differences in the overall neuronal density in L1 and L2 and/or neuronal size in L3 (Polgár, Thomson, Maxwell, Al‐Khater, & Todd, 2007; Abraira et al., 2017 for review see Ribeiro‐Da‐Silva & De Koninck, 2008; Peirs et al., 2020).

Choosing appropriate marker(s) is important for the identification of astrocytes (Zhang et al., 2019). We found a differential degree of somatic co‐localization of the prototypical astrocyte markers GFAP, S100B, and GS in astrocytes of the upper dorsal horn. The overall degree of co‐localization of only about 50% emphasizes the necessity for an educated selection of marker(s) and the subsequent interpretations in future studies. Interestingly, GS showed far less somatic co‐localization with both GFAP and S100B in L3 compared to L1 and L2; hinting for a potential higher need for the conversion of glutamate to glutamine in the nociceptive laminae L1 and L2. Glutamine is substrate for both glutamate and GABA. One might speculate that the transmission of pain‐related information might require a faster recycling of neurotransmitters than the processing of nonnociceptive information. Further, neurons in L1 and L2 are known to be mostly under tonic GABAergic inhibition whereas L3 is predominantly under glycinergic control (Takazawa et al., 2017; Takazawa & MacDermott, 2010), providing further explanation for the lamina‐specific need of GS.

We found a largely different expression profile for the 10 astrocyte‐enriched proteins of interest across the upper dorsal horn. These differences cannot simply be explained by the observed differences in astrocyte density since S100B, GS, NDRG2 and GLT1 were uniformly expressed across all laminae and Cx30 and Kir4.1 were expressed to a lesser extent in L1 as compared to L2 (and L3; for Kir4.1). Notably, the prominent astrocyte proteins GFAP, Cx43, GLAST, and AQP4 were all expressed at significantly higher levels in the superficial laminae L2 and/or L1 compared to L3. These proteins are hallmarks of astrocytic function representing their main tasks: structural support, network connectivity and glutamate as well as glymphatic system‐mediated clearance, respectively. These data suggests that integration of nociceptive information from the periphery requires astrocytes to provide more stability, to be better connected and better equipped for clearance of neurotransmitters, ions and waste compared to astrocytes integrating nonnociceptive information in deeper laminae.

Caution is required since fibrous astrocytes in the white matter bordering L1 could be contributing to the measured intensity levels since fibrous astrocytes are known to express GFAP to a higher extent than protoplasmic astrocytes (Miller & Raff, 1984). But since GFAP is an important structural protein of astrocytes, the abundance in L1 might also stem from a pronounced need for stability in delineating grey from white matter tissue. Further, the level of GFAP is known to influence glutamate transporter trafficking in astrocytes (Hughes, Maguire, McMinn, Scholz, & Sutherland, 2004). A higher turnover of glutamate in the superficial laminae L1 and L2 could be accompanied by an increased trafficking of glutamate transporters requiring higher levels of GFAP compared to astrocytes in L3.

Astrocytes are pivotal for glutamate homeostasis. They not only enable uptake of glutamate after neuronal activity via the glutamate transporter GLAST and GLT1 but also fuel the system by conversion of glutamate to glutamine through the glutamine synthetase (for review see Mahmoud et al., 2019). Reported differences in the expression profile of the glutamate transporter across the spinal cord hinted at regional adaptations (Rothstein et al., 1994; Tao, Gu, & Stephens Jr., 2005) and were reproduced and extended here. Dynamic linkage of neuronal activity and astrocytic transporter function could be portrayed by a higher GLAST expression and also GS‐co‐localization in the nociceptive laminae L1 and L2 compared to L3. Activation of high‐threshold nociceptive fibers is also linked to higher glutamate levels compared to activation of low‐threshold fibers (Al‐Ghoul et al., 1993; Kangrga & Randic, 1991). Swift and efficient clearance of glutamate after neuronal activity is necessary for fast excitatory synaptic transmission and to prevent excitotoxicity. The higher expression levels of GLAST in L1 and L2 compared to L3 might minimize synaptic spill over by enhanced levels of glutamate after activation of high‐threshold sensory fibers. This indicates that astrocytes are adapted to the specific needs of synaptic transmission in nociceptive laminae.

The water channel AQP4 has been shown to be highly expressed in the apex of the dorsal horn (Oklinski et al., 2014; Oklinski et al., 2016; Oshio et al., 2004; Vitellaro‐Zuccarello, Mazzetti, Bosisio, Monti, & De Biasi, 2005). In this study, AQP4 was found to be higher expressed in L1 compared to L3. Either vascularization is more pronounced in the nociceptive laminae or the astrocytic expression is higher (or both) allowing a faster clearance of ions, water and waste in nociceptive circuits compared to tactile circuits.

Similarly, Cx43 expression seems to be adapted to the differing needs of the nociceptive and the tactile circuits within the upper dorsal horn. The nociceptive circuits could demand astrocytes to work more efficiently than astrocytes in the tactile circuit by forming more gap junctions. This hypothesis is supported by a greater real‐time coupling speed of astrocytes residing in the superficial laminae L1 and L2 compared to astrocytes in L3. This difference in coupling speed cannot simply be explained by the different astrocyte density in these laminae since the somatic distance was corrected for and second‐degree coupling at greater distances was not included. The faster coupling speed is in line with the increased Cx43 expression in the nociceptive L1 and L2 compared to L3. Furthermore, Cx43 plays a role in the development of central neuropathic pain and chronic pain states highlighting its prominent role in nociceptive circuits (Chen et al., 2012). The role of Cx30 is less clear. To date, Cx30 seems to be not involved in the development of chronic pain conditions. However, the exact contribution of Cx30 to the coupling properties and network formation of spinal astrocytes remains to be elucidated. Whereas the connexins were differentially expressed, the overall network formation and qualities of spinal astrocytes were similar across all laminae tested facilitating information flow also across laminar borders. Understanding the contribution of the different connexins to the astrocytic network formation is important as the network function confers an electrical isopotentiality to the system hindering astrocytic membrane depolarization and maintaining the inward driving force for K^+^ ions (Huang et al., 2018; Ma et al., 2016).

The passive linearity in the electrophysiological profile of astrocytes stems from the dense expression of K^+^ channels (for review see Seifert, Henneberger, & Steinhäuser, 2018). The level of expression of certain K^+^ channels varies and potentially confers subtle differences to the electrophysiological profile and to the buffering of K^+^ ions after neuronal activity (Kofuji & Newman, 2004). Significant contribution to spatial buffering has been attributed to the inwardly rectifying Kir4.1 channel (Ransom & Sontheimer, 1995; Sibille, Dao, Holcman, & Rouach, 2015). A reduced Kir4.1 expression intensity in the superficial dorsal horn has been reported earlier (Olsen, Campbell, & Sontheimer, 2007) and was confirmed here. However, the observed differential expression pattern was not translated to the electrophysiological data. L1 of the dorsal horn was characterized by a significantly lower expression of Kir4.1 as compared to L3. But notably, astrocytes in L1 were also characterized by a greater effect of Kir4.1 blockade on passive membrane properties than astrocytes in L3. Since fluorescence intensity does not necessarily translate into efficiency, this could indicate that astrocytes in L1 express fewer Kir4.1 channels, but those expressed contribute more to the K^+^ conductance. Noxious stimuli can raise the extracellular K^+^ levels to a greater extent than innocuous signals (Heinemann et al., 1990). Further, the activity of nociceptive C‐fibers is highly sensitive to the level of extracellular K^+^ (Asai, Kusudo, Ikeda, & Murase, 2002; Asai, Kusudo, Ikeda, Takenoshita, & Murase, 2002). In general, without stimulation, the levels of extracellular K^+^ are lower in the superficial laminae compared to deeper laminae (Heinemann et al., 1990; Svoboda, Motin, Hajek, & Syková, 1988); further suggesting a differential contribution of K^+^ channels. Once more indicating that nociceptive signals might require a more efficient clearance compared to nonnociceptive signals. Nevertheless, the overall effect of BA‐induced Kir4.1 blockade was relatively small in all laminae; questioning the relative importance of Kir4.1 or highlighting the limitations of voltage‐clamping astrocytes in particular. The lack of pharmacological tools to selectively block K^+^ channels further makes it challenging to investigate the individual role of different K^+^ channels expressed in astrocytes. Furthermore, if the astrocyte RMP is dominated by K^+^ conductance, then one expects that blocking a fraction of the conductance with BA should not change the RMP markedly as the remaining K^+^ conductance is sufficient to keep the membrane potential near the K^+^ equilibrium potential. Our data are consistent with this view and imply the relative lack of a dominant conductance with an equilibrium potential positive to that of the K^+^ Nernst potential.

The highly negative RMP of astrocytes enables them to efficiently uphold their homeostatic functions by maintaining the respective driving force needed. Astrocytes located in L3 had a minor but significantly more depolarized RMP relative to astrocytes located in L2. One potential explanation might be the reduced responsiveness of astrocytes in L3 to Kir4.1 blockade. Kir4.1 is handled as one contributor to the hyperpolarized RMP of astrocytes and BA‐induced blockade of Kir4.1 channels had no effect on the RMP of astrocytes in L3.

All astrocytic tasks are coordinated by a complex spectrum of intracellular calcium elevations mediated by a diverse range of pathways providing the relevant spatial and temporal encoding of the presented information (Agulhon et al., 2008). In appreciation of the complexity of astrocytic calcium signaling, the analysis in this study was limited to the somatic calcium event rate by choice. The overall event rate was similar across the laminae and we found that the event rate of spontaneous calcium signals in spinal astrocytes was also not affected by bath application of TTX. This independence of neuronal action potential firing is in line with observations in other CNS regions (Chai et al., 2017). However, it has to be taken into consideration that under TTX spontaneous vesicle fusion is still possible and spontaneous neurotransmitter release from neurons may, to some degree, still contribute to spontaneous somatic calcium signals in astrocytes.

In summary, the present results paint the picture of spinal astrocytes capable of fine‐tuning their features and properties to the requirements of their neuronal environment. The differences were observed over a small area extension, thereby highlighting the existence of lamina‐specific heterogeneity of astrocytes. Deciphering the neuronal elements of spinal circuitries alone has not provided a full picture of spinal synaptic transmission, its adaptations and importantly its potential malfunctions. We have to overcome the temptation of treating astrocytes as a homogenous cell population and have to appreciate the complexity that lies within. The data reported herein pave the way for detailed functional and molecular studies of dorsal horn astrocytes, and explorations of their impact on neuronal function, to commence.

## CONFLICT OF INTEREST

The authors declare no conflict of interest.

## AUTHOR CONTRIBUTIONS

Mira T. Kronschläger, Baljit S. Khakh, and Jürgen Sandkühler designed the research. Mira T. Kronschläger, Anna S. M. Siegert, Felix J. Resch, and Pradeep S. Rajendran generated and/or analyzed the data. Mira T. Kronschläger, Baljit S. Khakh, and Jürgen Sandkühler wrote the article. All authors agreed to the final version of the article.

## Supporting information


Movie S1

Movie S2

Movie S3

## Data Availability

Raw data is available on request from the authors.
